# Csmd2 Is a Synaptic Transmembrane Protein that Interacts with PSD-95 and Is Required for Neuronal Maturation

**DOI:** 10.1523/ENEURO.0434-18.2019

**Published:** 2019-05-07

**Authors:** Mark A. Gutierrez, Brett E. Dwyer, Santos J. Franco

**Affiliations:** 1 Department of Pediatrics, University of Colorado School of Medicine, Aurora, CO 80045; 2 Cell Biology, Stem Cells and Development Graduate Program, University of Colorado School of Medicine, Aurora, CO 80045; 3 Program of Pediatric Stem Cell Biology, Children’s Hospital Colorado, Aurora, CO 80045

**Keywords:** Csmd2, CUB, dendrite, dendritic spine, Sushi, synapse

## Abstract

Mutations and copy number variants of the CUB and Sushi multiple domains 2 (*CSMD2*) gene are associated with neuropsychiatric disease. *CSMD2* encodes a single-pass transmembrane protein with a large extracellular domain comprising repeats of CUB and Sushi domains. High expression of *CSMD2* in the developing and mature brain suggests possible roles in neuron development or function, but the cellular functions of CSMD2 are not known. In this study, we show that mouse *Csmd2* is expressed in excitatory and inhibitory neurons in the forebrain. Csmd2 protein exhibits a somatodendritic localization in the neocortex and hippocampus, with smaller puncta localizing to the neuropil. Using immunohistochemical and biochemical methods, we demonstrate that Csmd2 localizes to dendritic spines and is enriched in the postsynaptic density (PSD). Accordingly, we show that the cytoplasmic tail domain of Csmd2 interacts with synaptic scaffolding proteins of the membrane-associated guanylate kinase (MAGUK) family. The association between Csmd2 and MAGUK member PSD-95 is dependent on a PDZ-binding domain on the Csmd2 tail, which is also required for synaptic targeting of Csmd2. Finally, we show that knock-down of *Csmd2* expression in hippocampal neuron cultures results in reduced complexity of dendritic arbors and deficits in dendritic spine density. Knock-down of *Csmd2* in immature developing neurons results in reduced filopodia density, whereas *Csmd2* knock-down in mature neurons causes significant reductions in dendritic spine density and dendrite complexity. Together, these results point toward a function for Csmd2 in development and maintenance of dendrites and synapses, which may account for its association with certain psychiatric disorders.

## Significance Statement

Variants in the CUB and Sushi multiple domains (*CSMD*) genes have been associated with neuropsychiatric disorders that negatively affect cognitive and social performance. However, the mechanisms by which CSMD proteins contribute to proper brain function have yet to be understood. This study demonstrates that mouse Csmd2 is a synaptic protein that interacts with synaptic scaffold protein postsynaptic density (PSD)-95. We also determine that Csmd2 is required for the development and maintenance of the dendritic arbor and dendritic spines of neurons. These results indicate that Csmd2 participates in the development and maintenance of synapses in the mammalian forebrain. Perturbation or loss of Csmd2 function could result in pathologic conditions associated with neuropsychiatric disease.

## Introduction

Neurologic disorders such as schizophrenia, autism spectrum disorder, and Alzheimer’s disease are characterized by deficits in cognitive and social abilities that significantly affect an individual’s quality of life. It is widely hypothesized that these disorders are the result of defects in the capacity of neurons to establish proper connections within neural circuits. These deficits are observed in the contexts of neuronal migration, dendrite development, and synapse formation in the developing cerebral cortex (Fukuda and Yanagi, 2017; Martinez-Cerdeno, 2017). Such defects would affect the function of the neural circuits that give rise to an individual’s higher-order cognitive abilities, such as learning and memory. However, the molecular mechanisms that lead to the onset of cognitive disorders remain to be fully understood.

A number of genome-wide association studies focusing on copy-number variants and single nucleotide polymorphisms have identified novel risk factors for psychiatric disorders. Deletions in members of the CUB and Sushi multiple domains (*CSMD*) gene family have been implicated in the occurrence of autism spectrum disorder, schizophrenia, and other neurodevelopmental disorders associated with deficits in cognitive ability and alterations in behavior (Havik et al., 2011; Donohoe et al., 2013; Steen et al., 2013; Koiliari et al., 2014; Sakamoto et al., 2016; Shi et al., 2017). The three *CSMD* genes, *CSMD1-3*, encode proteins that are single-pass transmembrane molecules with very large extracellular domains and short cytoplasmic tails (Lau and Scholnick, 2003). The CSMD genes are expressed strongly in the brain, but very little is known about the cellular functions of CSMD proteins. Their extracellular domains comprise multiple repeats of CUB (Clr/Cls, epidermal growth factor related sea urchin protein, and bone morphogenetic protein 1) and Sushi domains, which is a shared feature of several proteins that regulate dendrite development and synapse function (Gally et al., 2004; Zheng et al., 2004, 2006; Walker et al., 2006; Gendrel et al., 2009; Tang et al., 2011, 2012; Wang et al., 2012; Fisher and Mott, 2013). Additionally, Csmd1 has been previously identified in a proteomic screen as a protein localized to forebrain synapses using proximity biotinylation of synaptic cleft proteins (Loh et al., 2016). This suggests a synapse-specific role of the CSMD protein family in cellular function. However, the cellular functions of the CSMD proteins have yet to be reported.

Here, we characterized the expression, localization, associations, and functions of Csmd2 in the mouse forebrain. We found that *Csmd2* mRNA and Csmd2 protein are expressed in excitatory and inhibitory neurons in the mouse neocortex and hippocampus. Using biochemical methods to probe different membrane fractions of mouse brain homogenates, we found that Csmd2 was enriched in synaptosome-containing fractions, particularly in the postsynaptic density (PSD). We further validated these findings by immunohistochemistry, showing that Csmd2 localizes to dendritic spines where it colocalizes with the postsynaptic scaffold protein PSD-95. Utilizing yeast two-hybrid screening as well as co-immunoprecipitation assays, we found that the intracellular tail domain of Csmd2 interacts with PSD-95. This interaction depends on the PDZ-binding motif on Csmd2, and mutation of this PDZ ligand abolished Csmd2 interaction with PSD-95 and its synaptic enrichment. Finally, shRNA-mediated knock-down of *Csmd2* in cultured hippocampal neurons resulted in reduced dendritic filopodia in immature cells and eventually decreased dendrite complexity and dendritic spine density as neurons matured. Later knock-down of *Csmd2* in mature hippocampal neurons resulted in similarly reduced dendritic spine density and reduced dendrite complexity. Taken together, these results indicate that Csmd2 is a transmembrane protein localized to dendrites and synapses in the brain, and is required for the development and maintenance of dendrites and dendritic spines. This suggests a role for Csmd2 in synaptic development and function that may be perturbed in certain neuropsychiatric disorders.

## Materials and Methods

### Animals

Animals were maintained according to the guidelines from the Institutional Animal Care and Use Committee of the University of Colorado School of Medicine. All experiments involving mouse tissue were conducted using hybrid F1 mice resulting from crosses between *129 × 1/SvJ* (https://www.jax.org/strain/000691, RRID: MGI:5653118) and *C57BL/6J* (https://www.jax.org/strain/000664, RRID: MGI:5656552). Mice of either sex that resulted from these crosses were used in this study.

### Quantitative real-time PCR analysis

**Figure 1.**
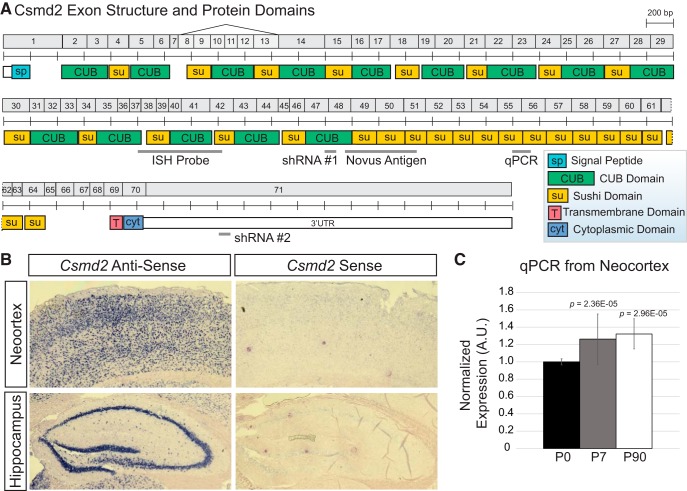
*Csmd2* mRNA is expressed in the mouse forebrain. ***A***, Schematic of numbered exons of the mouse *Csmd2* gene and domain structure of mouse Csmd2 protein, noting locations of alternative mRNA splicing, probes used for *in situ* hybridization and quantitative PCR analysis, antigen used to generate the anti-Csmd2 antibody from Novus, and target locations of Csmd2 shRNAs. ***B***, *In situ* hybridization showed broad expression of *Csmd2* mRNA throughout all neuronal layers in the neocortex and hippocampus. A sense-strand probe was used as a negative control. ***C***, Quantitative PCR analysis showed a slight increase in *Csmd2* mRNA expression in the neocortex from time point P0 to P7 and P90. Values were normalized to Cyclophilin A expression and graphed (average ± SEM of biological replicates) relative to the P0 time point. A.U., arbitrary units.

Total RNA was isolated from mouse cerebral cortices at the timepoints indicated in Figure 1*C* using the QIAGEN RNeasy Mini kit (QIAGEN, 74104) according to the manufacturer’s recommended instructions. RNA yields of each sample were quantified by an Eppendorf BioSpectrometer Basic apparatus. cDNA was reverse transcribed from 500-ng total RNA using the iScript cDNA Synthesis kit (Bio-Rad, 1708891). Reactions were performed in an Eppendorf MasterCycler EP Gradient 96-well thermal cycler according to the recommended instructions provided by the Bio-Rad iScript cDNA Synthesis kit. Real-time quantitative PCR analysis was performed using a Bio-Rad CFX Connect Real-Time PCR Detection System. Acquisition of data were then performed on a Bio-Rad CFX Manager software. Each PCR comprised of both forward and reverse primers each at a concentration of 400 nM with 1 μl of cDNA diluted five-fold, 7.4 μl of nuclease-free water, and 10 μl of iQ SYBR Green Supermix (Bio-Rad, 170-8880). Relative expression of *Csmd2* was assessed using the Δ-Ct method against mRNA for the housekeeping gene *Cyclophilin A*. Csmd2 primers: forward (5’AGTGCAACCACGGCTTCTA-3’) and reverse (5’GGCCACAGGACACCAAGA3’). Cyclophilin A primers: forward (5’ACGCCACTGTCGCTTTTC3’) and reverse (5’ACCCGACCTCGAAGGAGA3’).

### Immunohistochemistry

Mouse brains were fixed by transcardial perfusion with 4% paraformaldehyde before dissection and additional postfixation for 3 h at room temperature. Free-floating coronal sections were cut at 75 μm on a vibratome. Before immunohistochemical analyses, sections were subjected to antigen retrieval by incubation in 10 mM sodium citrate, pH 6.0 in a pressure cooker set to cook at pressure for 1 min.

For immunohistochemistry, sections and transfected cells were rinsed with 1× PBS twice for 5 min each. Samples were permeabilized and blocked with 10% normal donkey serum (Jackson ImmunoResearch, RRID: AB_2337254) and 0.1% Triton X-100 (Sigma-Aldrich) in 1× PBS for 1 h at room temperature. Primary antibodies: Csmd2 (Santa Cruz Biotechnology D18, RRID: AB_1562233 and G19, RRID: AB_1562234; Novus Biologicals, RRID: AB_11019509) 1:200; Ctip2 (Abcam, RRID: AB_2064130) 1:1000; Satb2 (Abcam, RRID: AB_2301417) 1:1000; and parvalbumin (Swant, RRID: AB_10000343) 1:500; Somatostatin (Millipore, RRID: AB_2255365) 1:250. Sections were incubated in primary antibodies overnight at room temperature. After washing samples with 1× PBS three times for 10 min each, relevant Alexa Fluor-conjugated, highly cross-adsorbed secondary antibodies made in goat (Life Technologies, 1:500 in PBS) were then applied to the sections for 1 h at room temperature. After rinsing with 1× PBS three times for 10 min each, 300 nM DAPI (Invitrogen, D1306) in 1× PBS was applied for 1 min. Coverslips were then applied to the sections with ProLong Diamond antifade reagent (Invitrogen, P36970). Samples were imaged using a Zeiss LSM 780 confocal microscope.

### DNA plasmid constructs

Partial gene fragments of mouse *Csmd2* cDNA were amplified using reverse-transcription polymerase chain reaction from total RNA extracted from adult mouse cerebral cortex. Remaining fragments were synthesized and purchased from Integrated DNA Technologies (IDT) as gBlocks. The full-length and 15x *Csmd2* cDNAs were cloned using NEBuilder HiFi (New England Biolabs, E2621) into an expression vector comprising a CMV promoter and chicken beta-actin enhancer (CAG), the preprotrypsin (PPT) leader sequence and three tandem FLAG epitopes (3xFLAG). Each expression construct was cloned so that the PPT leader sequence and 3xFLAG were fused in frame at the N terminus of Csmd2.

Mutation of the C-terminal PDZ-binding domain was achieved by synthesizing the mutant cytoplasmic domain as a gBlock and cloning into the wild-type constructs.shRNA plasmids contained both an shRNA expression cassette and a reporter gene expression cassette. The shRNA sequences were synthesized as gBlocks (IDT) and cloned downstream of a U6 promoter. shRNA sequences were: non-targeting control, 5’GCGATAGCGCTAATAATTT3’; Csmd2 shRNA #1, 5’ GGCAAAGTCCTCTACTGAA3’; Csmd2 shRNA #2, 5’GGACGTTCTTCAGATATAA3’. The reporter gene encoded a myristoylated form of TdTomato that targets the fluorescent reporter to the plasma membrane, which was cloned downstream of the CAG promoter/enhancer. All constructs were confirmed by DNA sequencing. Detailed methods and maps for all expression vectors will be provided on request.

### Synaptosomal fractionation

Preparation of synaptosomal fractions from mouse forebrain homogenate was performed as previously described (Dunkley et al., 2008). Mouse brain homogenates were subjected to separation via ultracentrifugation over a Percoll (GE Healthcare, 17-0891-01) gradient. The samples obtained from the fractions produced by this protocol were subjected to SDS-PAGE and Western blotting. Crude synaptosomal membranes (P2 pellets) and crude PSD fractions (TxP) were prepared as previously described (Sanderson et al., 2012).

### Western blotting

Protein concentrations were measured with the BCA assay (Pierce, Thermo Scientific, 23225) before SDS-PAGE and Western blot analysis. All protein samples were subjected to SDS-PAGE using 4–15% polyacrylamide gradient gels (Bio-Rad, 4561086). For cell lysates, 20–30 μg was loaded, while for synaptosomal fraction samples 40 μg of protein for each sample was loaded on the gels. Separated proteins were then electroblotted using a TransBlot Turbo system to TransBlot Turbo Mini-size PVDF membranes (Bio-Rad, 1704272). Membranes were subsequently blocked with 1× TBS containing 0.1% Tween 20 (1× TBST) with 5% (w/v) blotting-grade blocker (Bio-Rad, 1706404) and probed with the primary antibody of interest diluted in 1× TBS containing 0.1% Tween 20 and 0.5% blocker at room temperature overnight. Primary antibodies for mouse Csmd2 were used at a dilution of 1:500, PSD-95 (ThermoFisher, RRID: AB_325399) at 1:1000, and DYKDDDK (FLAG; ThermoFisher, RRID: AB_2536846) at 1:500. Membranes were washed three times in 1× TBST for 10 min each before 1 h of incubation at room temperature with horseradish peroxidase (HRP)-conjugated secondary antibodies used at 1:10 000. Membranes were visualized using the Clarity Western ECL Blotting Substrates (Bio-Rad, 1705060) according to the manufacturer’s recommended instructions in a Bio-Rad Chemidoc Universal Hood III imaging system.

### *In utero* electroporation

*In utero* electroporations were performed as described (Franco et al., 2011). Briefly, timed pregnant mice [embryonic day (E)15.5] were anesthetized and their uterine horns exposed. One to two microliters of endotoxin-free plasmid DNA was injected into the embryos’ lateral ventricles at 1 mg/mL each. For electroporation, five pulses separated by 950 ms were applied at 50 V. To target the hippocampus, electrodes were placed in the opposite orientation compared to targeting the neocortex. Embryos were allowed to develop *in utero* and then postnatally until the indicated time.

### Yeast two-hybrid analysis

Yeast two-hybrid screening was performed by Hybrigenics Services, S.A.S. Details on this service can be found on the Hybrigenics Ultimate Y2H webpage (http://www.hybrigenics-services.com/). The mouse Csmd2 cytoplasmic domain (amino acids 3557-3611) was used as the bait protein and a mouse adult brain cDNA library (ref: [AMB]) was the prey. For each interaction, a predicted biological score was computed to assess the interaction reliability (Rain et al., 2001). This score represents the probability of an interaction to be non-specific: it is an e-value, primarily based on the comparison between the number of independent prey fragments found for an interaction and the chance of finding them at random (background noise). The value varies between 0 and 1. Several thresholds have been arbitrarily defined to rank the results in four categories from A (the highest confidence rank) to D (Formstecher et al., 2005). Complete results of the yeast two-hybrid screen can be found as Extended Data Figures 8-1, 8-2.

10.1523/ENEURO.0434-18.2019.f8-1 Extended Data Figure 8-1 **Complete Yeast 2-Hybrid Results Summary**. For each interaction, a Predicted Biological Score (PBS) is computed to assess the interaction reliability. This score represents the probability of an interaction to be non-specific: it is an e-value, primarily based on the comparison between the number of independent prey fragments found for an interaction and the chance of finding them at random (background noise). The value varies between 0 and 1. Several thresholds have been arbitrarily defined in order to rank the results in 4 categories from A (the highest confidence rank) to D. PBS D generally represents interactions identified through one unique prey fragment or multiple identical ones. It can be interactions hardly detectable by the Y2H technique (low representation of the mRNA in the library, prey folding, prey toxicity in yeast) or it can be false-positive interactions. The PBS is adjusted by integrating the PBS of other interactions from the database in which interaction domains of the involved proteins have been found. For example, reciprocal interactions found in independent screens are technically very reliable and thus tagged as A, B or C. Two additional categories have been implemented: PBS E and PBS F. The PBS E represents interactions involving prey domains connected to more than 10 different mouse bait proteins in the entire database. This arbitrary threshold allows flagging of highly – or relatively highly – connected protein domains. Experimentally proven artifacts of the Y2H technology are flagged with a PBS F. These can be LexA or Gal4 protein binders or binders of the DNA sequence upstream of the reporter gene. Figure 8-1, PDF file.

**Figure 2.**
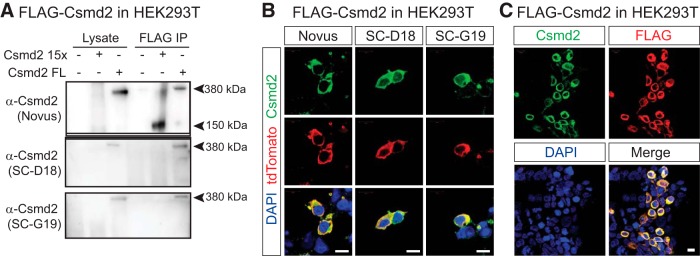
Validation of Csmd2 antibodies. ***A***, HEK293T cells were transfected with expression constructs for FLAG-tagged full-length (Csmd2 FL) or truncated (Csmd2 15x) Csmd2. The Novus α-Csmd2 antibody detected both full-length and truncated forms in Western blot analysis from whole cell lysates or after immunoprecipitation with α-FLAG antibody. SC-D18 and SC-G19 α-Csmd2 antibodies only detected the full-length construct. ***B***, Fluorescence immunocytochemistry of HEK293T cells co-transfected with FLAG-Csmd2 full-length and myristoylated-tdTomato as a transfection marker. All three antibodies recognized exogenous Csmd2 in the transfected cells. ***C***, As in ***B*** but stained with anti-FLAG antibody. Csmd2 (SC-G19) signal colocalized with FLAG signal at the plasma membrane. Scale bars, 10 µm.

10.1523/ENEURO.0434-18.2019.f8-2 Extended Data Figure 8-2 **Yeast 2-hybrid Prey Fragment Analysis**. Schematic representations of information on bait and prey structural, functional and interaction domains. Selected Interaction Domain (SID) is the amino acid sequence shared by all prey fragments matching the same reference protein. SIDs often correspond to an identified structural or functional domain. Figure 8-2, PDF file.

### 3xFLAG pull-down and co-immunoprecipitation

For FLAG pull-down and co-immunoprecipitation experiments, samples were lysed in a working solution of 50 mM Tris-HCl, 1 mM NaCl, 1% Triton X-100, and 1 mM EDTA, pH 7.6. Every 10 ml of this solution was supplemented with 1 cOmplete ULTRA, Mini, EDTA-free protease inhibitor cocktail tablet (Roche, 11836170001). After lysate pre-clearing, samples were incubated for 3 h at 4°C with anti-DYDDDDK Affinity Gel (Rockland, RRID: AB_10704031). For all other co-immunoprecipitation experiments, samples were lysed in the aforementioned lysis buffer. After lysate preclearing, samples were incubated with an antibody against the targeted protein of interest overnight at 4°C. After antibody binding, samples were incubated for 3 h at 4°C with Protein G Mag Sepharose Xtra (GE Healthcare Life Sciences, 28967066) beads. After incubation, washes were conducted according to the corresponding manufacturers’ recommended protocol. Samples were eluted from beads via incubation with Laemmli sample buffer (Bio-Rad, 1610737) at 37°C for 20 min before analysis by Western blotting.

### Primary hippocampal neuron culture

Primary cultured hippocampal neurons were prepared from E17.5 *C57Bl/6J* mice. Hippocampal tissue was manually dissected and dissociated as previously described (Lesuisse and Martin, 2002). A total of 500,000 cells were seeded per well onto poly-D-lysine-coated (Millipore, A-003-E) 12-mm cover slips in 24-well plates in DMEM (Corning, 10-017-CV) containing 10% fetal bovine serum (Gibco, 10437010) and 1% penicillin/streptomycin (Lonza, 17-602E). At 2 d *in vitro* (DIV), the DMEM-based culture medium was replaced with EMEM (Lonza, 12-125F) containing 2.38 mM sodium bicarbonate (Sigma, S5761-500G), 2 mM stabilized L-glutamine (Gemini Bio, 400-106), 0.4% glucose (Sigma, G7021-100G); 0.1 mg/ml apo-transferrin (Gemini Bio, 800-130P), 2% Gem21 NeuroPlex Serum-Free Supplement (Gemini Bio, 400-160), 5% fetal bovine serum (Gibco, A31604-01), and 1% penicillin/streptomycin (Lonza, 17-602E). At 3 DIV, half of the culture medium was replaced with fresh EMEM-based medium. At the indicated times, coverslips were fixed in 4% paraformaldehyde for 20 min at room temperature, washed three times with PBS and mounted onto microscope slides using ProLong Diamond antifade reagent (Invitrogen, P36970).

### Transfection of primary hippocampal neuron cultures

On dissection and dissociation of hippocampal tissue as described above, cells were transfected with the Amaxa Mouse Neuron Nucleofector kit (Lonza, VPG-1001) using the Amaxa Nucleofector II device (Lonza). Transfection was conducted according to the manufacturer’s recommended protocol for primary mouse hippocampal and cortical neurons. Matured neuronal cultures (14 DIV) were transfected using the Lipofectamine 2000 reagent (Invitrogen, 11668-019). Each transfection reaction was prepared as previously reported for the transfection of adherent primary neurons in a 24-well format (Dalby et al., 2004) with the modification of a 1 μg of total DNA used with 1 μl of Lipofectamine 2000 reagent for each 24-well plate holding 1 ml of cell culture media.

### Statistical analysis

Dendritic spine densities and morphologic analyses were performed using ImageJ. All quantitative data were graphed as the mean with the SEM of each experimental group. See Table 1 for the details of statistical analysis.

**Table 1.** Statistical table

Data structure	Type of test	Power/confidence interval
Figure 10*B*, assumed normal distribution	Two-tailed *t* test	95% CI
Figure 10*C*, assumed normal distribution (each sample data group at noted radius from soma)	Two-tailed *t* test	95% CI
Figure 10*D*, assumed normal distribution	Two-tailed *t* test	95% CI
Figure 11*B*, assumed normal distribution	Two-tailed *t* test	95% CI
Figure 12*B*, assumed normal distribution (each sample data group at noted radius from soma)	Two-tailed *t* test	95% CI
Figure 12*C*, assumed normal distribution	Two-tailed *t* test	95% CI

Structure of data and method of statistical analysis used to determine significance of features observed in the noted figures.

## Results

### *Csmd2* mRNA is expressed in the mouse neocortex and hippocampus

The mouse *Csmd2* gene comprises 71 exons and is predicted to encode a 13,555 base long mRNA (Fig. 1*A*). While cloning the full-length Csmd2 cDNA from postnatal forebrain, we also identified a splice variant in which exon 7 splices to exon 14 (Fig. 1*A*). The protein encoded by the full-length mRNA is predicted to be 3611 amino acids with an approximate molecular weight of 392 kDa. The TMHMM 2.0 Server (http://www.cbs.dtu.dk/services/TMHMM/; Krogh et al., 2001) predicts a single transmembrane helix in the mouse Csmd2 protein at amino acids 3534–3556 (Fig. 1*A*). Results from the TatP 1.0 Server (http://www.cbs.dtu.dk/services/TatP/; Bendtsen et al., 2005) prediction indicate the presence of a signal peptide in the N-terminal 37 amino acids of Csmd2, with a likely cleavage site between positions G37-R38 (Fig. 1*A*). The large extracellular domain of Csmd2 contains 14 complement C1r/C1s, Uegf, Bmp1 (CUB) domains, each separated by an intervening Sushi domain (Fig. 1*A*). Following the CUB/Sushi repeats is a series of 15 consecutive Sushi domains, the transmembrane domain and a cytoplasmic tail domain at the C terminus.

Publicly available databases show that human *CSMD2* mRNA (https://www.proteinatlas.org; Fagerberg et al., 2014) and mouse *Csmd2* mRNA (http://www.informatics.jax.org/expression.shtml; Diez-Roux et al., 2011) expression are highest in the central nervous system. To further analyze *Csmd2* mRNA expression in the adult mouse forebrain, we performed RNA *in situ* hybridization using a probe spanning exons 37–42 (Fig. 1*A*). We found *Csmd2* mRNA widely-expressed throughout the neuronal layers of the adult mouse neocortex and hippocampus (Fig. 1*B*). Quantitative real-time PCR analysis showed that *Csmd2* expression slightly increased in the neocortex during the first postnatal week, at which time it reached similar levels as in the adult (Fig. 1*C*). Together, these data indicate that mouse *Csmd2* encodes a large, single-pass transmembrane protein expressed in the developing and mature forebrain.

### Csmd2 protein is expressed in excitatory projection neurons and inhibitory interneurons

We next wanted to determine Csmd2 protein expression and localization in the forebrain. To this end, we first characterized several commercially-available antibodies against Csmd2. We generated cDNA expression plasmids for either full-length Csmd2, or a truncated form of Csmd2 in which the ectodomain contains only the 15 Sushi repeats proximal to the transmembrane domain (Csmd2 15x; Fig. 2*A*). Both constructs included a 3xFLAG tag at the N terminus, located just downstream of the signal peptide. On transfection of these constructs into HEK293T cells, Western blot analysis using three different anti-Csmd2 antibodies revealed the predicted 380-kDa band corresponding to full-length Csmd2, only in the transfected conditions (Fig. 2*A*). Immunoprecipitation of these samples using anti-FLAG beads before Western blotting confirmed that the bands in each condition corresponded to the exogenous FLAG-tagged Csmd2 protein (Fig. 2*A*). Only the anti-Csmd2 antibody from Novus was able to detect the truncated Csmd2 15x protein (Fig. 2*A*), indicating that the other 2 antibodies recognize more N-terminal regions of Csmd2. We further tested the antibodies by fluorescence immunocytochemistry on HEK293T cells co-transfected with full-length FLAG-Csmd2 together with myristoylated tdTomato (myr-tdTomato) as a transfection marker. We confirmed that all three antibodies labeled plasma membranes only in the transfected HEK293T cells, but not in untransfected cells (Fig. 2*B*). Furthermore, we confirmed colocalization of the FLAG tag and Csmd2 on the plasma membrane of transfected cells (Fig. 2*C*). These data indicate that all three antibodies can detect mouse Csmd2 protein in Western blot analyses and immunocytochemistry.

**Figure 3.**
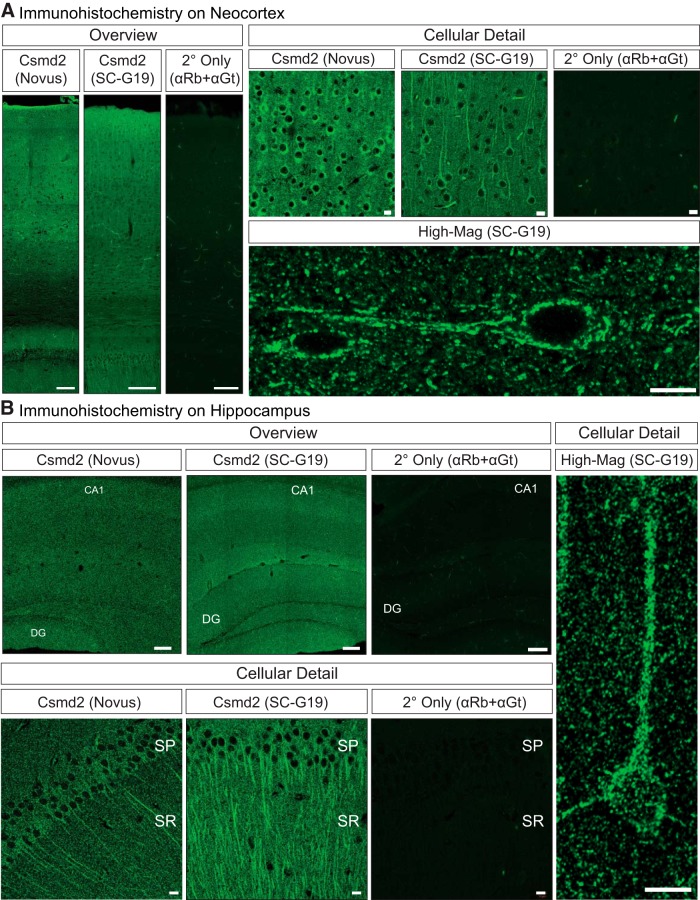
Detection of Csmd2 protein in the mouse forebrain. ***A***, Coronal section of adult mouse neocortex stained for α-Csmd2 (Novus or SC-G19) showed Csmd2 expression throughout neuronal layers of the neocortex. No significant signal was seen in the absence of primary antibody (α-goat plus α-rabbit Alexa Fluor-labeled secondary antibodies). Zoom-in images (right, cellular detail and high mag) show dendritic and somatodendritic distribution of Csmd2 and punctate patterns in the neuropil, as detected by Novus and SC-G19 α-Csmd2 antibodies. ***B***, Csmd2 expression in the adult mouse hippocampus appeared broad throughout the neuronal layers, as seen in the overviews (left). Zoom-in images (right, cellular detail and high mag) show somatodendritic patterns in cell bodies and punctate patterns in the neuropil. Scale bars: 100 μm (overview images) and 10 μm (cellular detail and high mag). CA1, cornu ammonis 1; DG, dentate gyrus; SP, stratum pyramidale; SR, stratum radiatum.

We next conducted fluorescence immunohistochemistry on coronal sections from adult mouse brains to determine Csmd2 protein localization in the forebrain. We observed Csmd2 signal distributed throughout the neocortex and hippocampus. In the neocortex, Csmd2 protein was detected throughout the neuronal layers, similar to localization of Csmd2 mRNA transcripts (Fig. 3*A*, overview). Control sections that were stained without primary antibodies (secondary antibodies only) displayed signal only in blood vessels, indicating that the staining pattern observed with the two Csmd2 antibodies represented widespread Csmd2 expression in the neocortex. Analysis at the cellular level revealed a somatodendritic pattern of Csmd2 expression in neocortical neurons, with additional punctate expression in the neuropil (Fig. 3*A*, cellular detail and high mag).

Similar to the neocortex, Csmd2 was widely expressed throughout the hippocampus (Fig. 3*B*, overview), in somatodendritic and punctate neuropil patterns (Fig. 3*B*, cellular detail and high mag). Higher magnification images of neurons in the CA1 layer using the SC-G19 antibody revealed that Csmd2 protein extended into the apical dendrites of neurons in the stratum pyramidale (Fig. 3*B*, high mag). In the stratum radiatum, Csmd2 signal was found in smaller puncta throughout the neuropil. Again, nearly all Csmd2 signal in the hippocampus was lost in the absence of a primary antibody (Fig. 3*B*). Taken together, these data indicate that Csmd2 is widely expressed throughout the mouse neocortex and hippocampus, exhibiting somatodendritic and punctate patterns within neurons.

**Figure 4.**
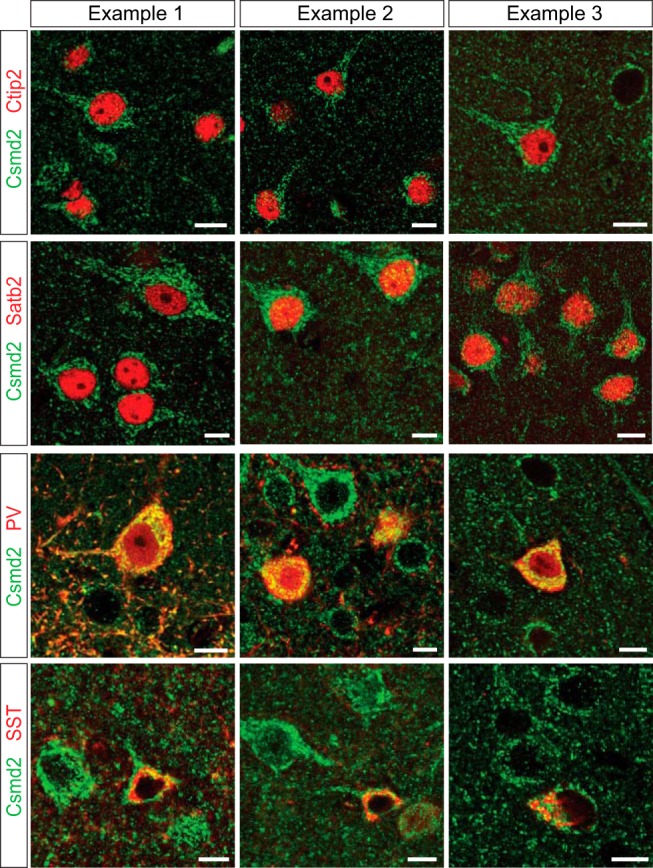
Csmd2 is expressed in multiple neuronal cell types. Coronal sections of adult mouse neocortex. Fluorescence immunohistochemistry revealed expression of Csmd2 (green) in Ctip2^+^ (red) and Satb2^+^ (blue) excitatory projection neurons, and in PV^+^ (red) and SST^+^ (red) inhibitory interneurons. Scale bars, 10 μm.

To elucidate which neuronal cell types express Csmd2, we probed adult mouse neocortical sections for Csmd2 (SC-G19 antibody) together with Ctip2 for excitatory corticofugal neurons, Satb2 for excitatory corticocortical projection neurons, and PV and SST for different inhibitory interneuron subtypes (Fig. 4). We observed Csmd2 expression in Ctip2^+^ and Satb2^+^ cells, demonstrating that Csmd2 is expressed in excitatory projection neurons. Additionally, Csmd2 was expressed even more robustly in PV^+^ and SST^+^ cells, indicating higher expression in inhibitory interneurons (Fig. 4). In each of these cases, Csmd2 exhibited a clear somatodendritic expression pattern. We also stained sections for Csmd2 together with markers for astrocytes (Aldh1l1) and oligodendrocytes (Olig2), but these stainings were inconsistent and inconclusive (data not shown), so it remains to be determined whether Csmd2 is expressed in neocortical or hippocampal glial cells. These data indicate that Csmd2 is expressed by both excitatory and inhibitory neurons, in the mouse forebrain.

### *Csmd2* is enriched in synapses

Genome-wide association studies have linked human *CSMD* genes with several psychiatric disorders (Shimizu et al., 2003; Floris et al., 2008; Glancy et al., 2009; Havik et al., 2011; Swaminathan et al., 2011). This raises the possibility that Csmd2 may participate in normal neuronal function in the brain. In support of this idea, several other CUB-containing and/or Sushi-containing proteins play roles in the development and function of dendrites or synapses. For example, LEV-10 and LEV-9 are *Caenorhabditis elegans* proteins that contain CUB and Sushi domains, respectively, and cooperate to regulate acetylcholine receptor function at neuromuscular junctions (Gally et al., 2004; Gendrel et al., 2009). The *C. elegans* CUB domain-containing proteins SOL-1 and SOL-2 are synaptic auxiliary proteins that modify the kinetics of AMPA-type ionotropic glutamate receptors (iGluRs; Zheng et al., 2004, 2006; Walker et al., 2006; Wang et al., 2012). Mammalian homologues of SOL-2, Neto1 and Neto2, contain two CUB domains and are key regulators of kainate-type and NMDA-type iGluRs (Ng et al., 2009; Zhang et al., 2009; Copits et al., 2011; Straub et al., 2011a,b; Tang et al., 2011, 2012; Fisher and Mott, 2012). The Sez6 family of proteins, which contain extracellular domains of multiple CUB and Sushi repeats similar to Csmd2, are critical for establishing normal dendritic arborization patterns and synaptic connectivity in the neocortex (Gunnersen et al., 2007). Interestingly, Csmd1 was recently identified in a proteomic screen as being localized to forebrain synapses using proximity biotinylation of synaptic cleft proteins (Loh et al., 2016). Therefore, we hypothesized that Csmd2 might play a role in dendrite and synapse development.

**Figure 5.**
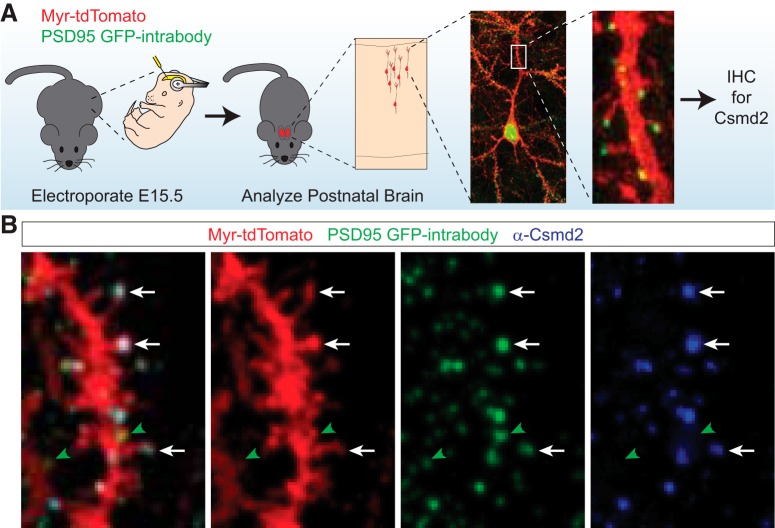
Csmd2 co-localizes with PSD-95 at synapses. ***A***, Schematic of experimental approach for *in vivo* labeling of neuronal dendritic spines with myrisotylated-tdTomato and PSDs with a GFP-fused intrabody targeting PSD-95. Electroporated brains were stained for Csmd2 (SC-G19) at P30. ***B***, Immunohistochemical analysis of P30 neurons after *in utero* electroporation showed localization of punctate Csmd2 at PSD-95^+^ synapses on both the dendritic shaft and at the ends of dendritic spines (white arrows). A subset of PSD-95^+^ puncta were not positive for Csmd2 (green arrowheads).

To begin to test this hypothesis, we used several complementary methods to determine if Csmd2 is localized to synapses in the mouse forebrain. First, we devised a strategy to help visualize individual synapses *in vivo*. We used *in utero* electroporation to introduce expression plasmids into progenitors of excitatory neurons in the cortex (Fig. 5*A*). We electroporated a myr-tdTomato construct together with an expression plasmid for a GFP-tagged intrabody targeting endogenous PSD-95 (iGFP-PSD-95; Gross et al., 2013), allowing us to visualize dendritic spines and PSDs of excitatory neurons in the mature cortex (Fig. 5*A*). When combined with Csmd2 immunohistochemistry, we readily found Csmd2 puncta co-localized with PSD-95 at the ends of dendritic spines (Fig. 5*B*, white arrows). Interestingly, not all PSD-95^+^ spines displayed detectable Csmd2 signal (Fig. 5*B*, green arrowheads), indicating some heterogeneity in the presence or levels of Csmd2 at spines.

**Figure 6.**
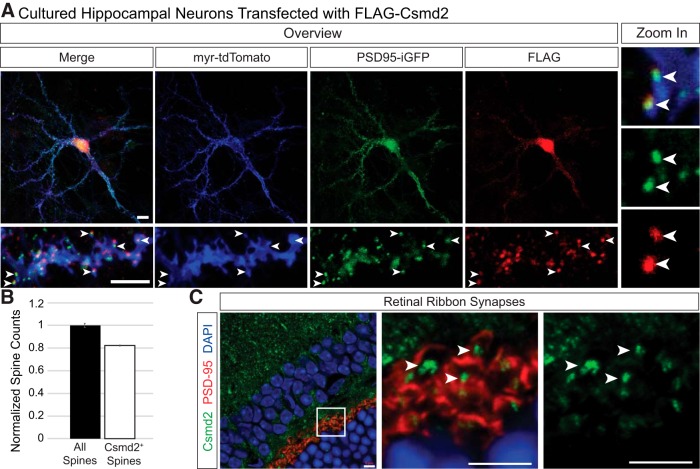
Csmd2 localizes to dendritic spines *in vitro* and to retinal ribbon synapses. ***A***, 21 DIV hippocampal neurons transfected with FLAG-Csmd2 showed somatodendritic α-FLAG staining (upper panels) and punctate expression throughout their dendrites, including in PSD-95^+^ dendritic spines (lower panels, arrowheads). Zoom-in images (right) showed colocalization of FLAG-Csmd2 with PSD-95. ***B***, Quantification (average ± SEM of biological replicates) of endogenous Csmd2 punctate expression revealed Csmd2 localization at >80% of dendritic spines at 14 DIV. ***C***, Fluorescence immunohistochemistry of P90 mouse retina revealed punctate Csmd2 expression in the synaptic layers, including at the center of ribbon synapses in the inner plexiform layer. Scale bars: 10 μm (***A***) and 5 μm (***C***).

We employed a similar approach to visualize Csmd2 localization in more detail in dissociated hippocampal neurons *in vitro*. We transfected a FLAG-tagged Csmd2 plasmid into E17.5 dissociated hippocampal neurons, together with iGFP-PSD-95 to visualize PSDs and myr-tdTomato as a transfection and membrane marker. We then performed immunocytochemistry for the FLAG tag at 21 DIV, at which point we could detect FLAG-Csmd2 colocalized with PSD-95^+^ puncta (Fig. 6*A*). To further analyze endogenous Csmd2 localization more quantitatively, we labeled dissociated hippocampal neurons with myr-tdTomato at the day of harvest, E17.5, and then prepared primary hippocampal neuron cultures. Fluorescence immunocytochemistry probing for Csmd2 at 14 DIV revealed that ∼80% of labeled spines contain some Csmd2 signal (Fig. 6*B*). To test for synaptic localization in a third approach, we performed immunohistochemistry on P90 mouse retinal sections (Fig. 6*C*). We found Csmd2 localized throughout the retinal layers, including in a somatodendritic pattern in the inner nuclear layer and in punctate patterns in both the inner and outer plexiform layers. Higher magnification images revealed Csmd2 concentrated at the center of PSD-95^+^ ribbon synapses in the outer plexiform layer (Fig. 6*C*). Together, these data indicate that Csmd2 localizes to the soma, dendrites and at least a subset of synapses in multiple neuronal cell types.

**Figure 7.**
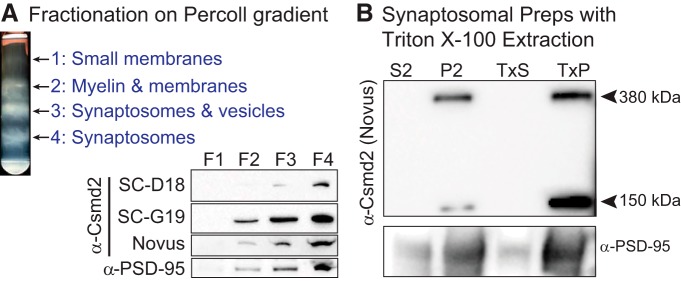
Csmd2 is found in synaptosomal and postsynaptic fractions. ***A***, Membrane fractionation of P30 mouse forebrain lysate using a Percoll gradient. Representative membrane fractions are shown at top. Equal amounts of protein from each fraction were analyzed by Western blot analysis using three different α-Csmd2 antibodies and an α-PSD-95 antibody. All three Csmd2 antibodies detected Csmd2 enriched in the synaptosome-containing fractions, along with PSD-95. ***B***, Preparation of crude synaptosomes showed a similar enrichment of Csmd2 and PSD-95 in the syaptosomal fraction (P2 pellet) compared to the soluble fraction (S2). Further extraction of P2 with Triton X-100 showed Csmd2 enriched in the PSD fraction (TxP pellet) with PSD-95, compared to the Triton X-100-soluble fraction (TxS). The Novus antibody detected full-length Csmd2 and a smaller band of unknown identity at ∼150 kDa.

To further characterize the subcellular localization of Csmd2 in forebrain neurons, we isolated synaptosomal fractions from P30 mouse whole brain tissue using a Percoll gradient (Dunkley et al., 2008), which allows for the separation of small membranes, myelin, membrane vesicles and synaptosomes (Fig. 7*A*). We ran equal amounts of protein from each fraction on an SDS-PAGE gel. On Western blot analysis of the fractions, we observed enrichment of Csmd2 in synaptosome-containing fractions F3 and F4, along with PSD-95 (Fig. 7*A*). Csmd2 was detected in synaptosomal fractions by all three Csmd2 antibodies tested. To determine in which compartment of the synaptosome Csmd2 was localized, we used a second method for the fractionation of the PSD from a crude synaptosomal preparation (Sanderson et al., 2012). We confirmed by this method that Csmd2 was found in the synaptosomal pellet (P2) fraction, specifically in the Triton X-100-insoluble PSD pellet fraction (TxP; Fig. 7*B*). Using this method, we also identified a smaller band at ∼150 kDa that was recognized by the Csmd2 Novus antibody (Fig. 7*B*). Although we have not yet identified this protein, it is possible that it may represent a cleavage product of the extracellular domain, or an alternative splice isoform that our RT-PCR assays did not detect. Together, these data show that Csdm2 is localized to synapses in the neocortex, hippocampus and retina.

### Csmd2 interacts with synaptic scaffold proteins

**Figure 8.**
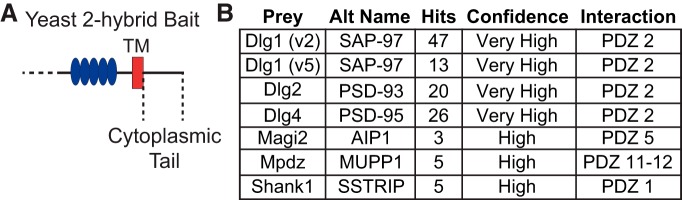
Candidate Csmd2 intracellular interaction partners. ***A***, Schematic of Csmd2 C-terminal end, showing the region of cytoplasmic tail used as bait for a yeast two-hybrid screen with an adult mouse brain cDNA library as prey. ***B***, Results of the two-hybrid screen revealed high-confidence hits with several synaptic scaffolding proteins. See also Extended Data Figure 8-1 for complete results of the screen. All interactions were mapped to specific PDZ domains within these multi-PDZ proteins. See also Extended Data Figure 8-2 for domain mapping of the interactions.

**Figure 9.**
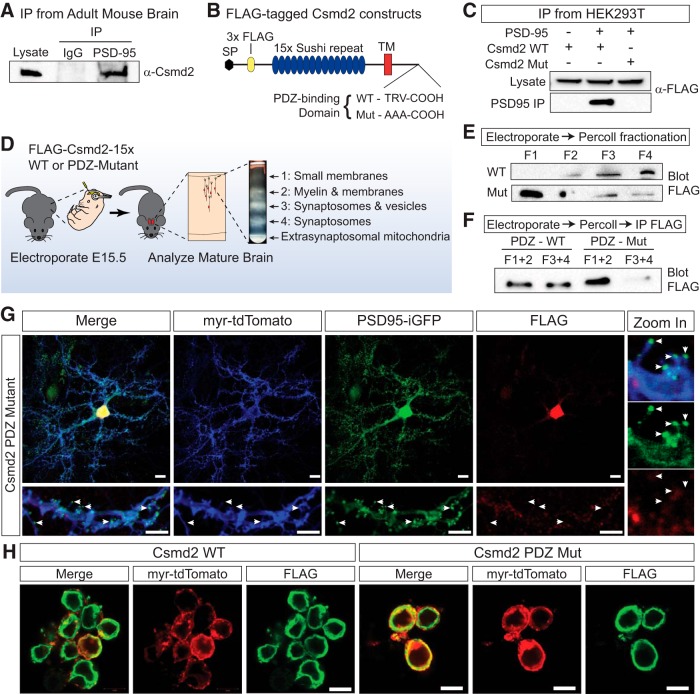
Csmd2 interacts with PSD-95 via a PDZ-binding domain. ***A***, Endogenous Csmd2 from adult mouse brain lysates co-immunoprecipitated with PSD-95, but not with control IgG. ***B***, Schematic of truncated, FLAG-tagged Csmd2 expression constructs used in ***C–F***. In the mutated construct (Mut), the PDZ-binding motif (TRV) was mutated to AAA. ***C***, Constructs from ***B*** were transfected into HEK293T cells with or without PSD-95 cDNA and lysates were immunoprecipitated with α-PSD-95. FLAG-Csmd2 with a WT, but not Mut, PDZ domain co-immunoprecipitated with PSD-95. ***D***, Schematic of experimental design for ***E***, ***F***. Constructs from ***B*** were electroporated into the neocortex at E15.5, and the electroporated region was subsequently microdissected from adult brains and fractionated on a Percoll gradient before Western blotting (***E***) or IP (***F***). ***E***, Equal amounts of protein from each fraction were run on an SDS-PAGE gel and Western blotted with α-FLAG. Csmd2 WT was enriched in synaptosomal fractions F3-F4, but Mut is found primarily in non-synaptosomal fractions F1-F2. ***F***, Similar experiment as in ***E***, but fractions were pooled in pairs and immunoprecipitated with α-FLAG affinity gel before SDS-PAGE and Western blot analysis. The Mut protein was lost from synaptosomal fractions F3-F4. ***G***, A total of 21 DIV hippocampal neurons expressing Csmd2 PDZ Mut showed a primarily somatic distribution of FLAG-Csmd2, with no signal observed at dendritic spines. This is in contrast to the WT version shown in Figure 6*A*. ***H***, Both the WT and PDZ Mut FLAG-Csmd2 proteins were localized to the plasma membrane when expressed in HEK293T cells. Scale bars, 10 µm.

To begin to study the possible functions of Csmd2 in the brain, we identified some of the molecular associations with Csmd2. We employed a yeast two-hybrid system to screen candidate target proteins expressed in the adult mouse brain for interactions with the intracellular portion of Csmd2. Using the entire cytoplasmic tail of Csmd2 as bait protein and an adult mouse brain library as prey, our screen identified seven proteins that interacted with the Csmd2 cytoplasmic tail domain with high or very high confidence (Fig. 8; Extended Data Fig. 8-1). Interestingly, several of the identified interactors are known synaptic scaffolding proteins of the membrane-associated guanylate kinase (MAGUK) family, including SAP-97, PSD-93, and PSD-95. Each interaction mapped to a specific PDZ domain (Fig. 8; Extended Data Fig. 8-2). We found that Csmd2 contains a putative class I PDZ-binding motif (TRV-_COOH_) at the extreme C terminus of its cytoplasmic tail (Fig. 9*B*).

As a starting point to validate our two-hybrid results, we performed immunoprecipitation of PSD-95 from mouse adult brain lysates and found that endogenous Csmd2 co-immunoprecipitated with PSD-95 (Fig. 9*A*). Furthermore, when we co-expressed PSD-95 with the FLAG-tagged Csmd2 construct (Fig. 9*B*) in HEK293T cells, FLAG-Csmd2 co-immunoprecipitated on PSD-95 pull-down (Fig. 9*C*). To determine if the interaction between PSD-95 and Csmd2 is dependent on PDZ/PDZ-ligand interactions (Long et al., 2003), we generated a construct in which the Csmd2 PDZ-binding domain was mutated from TRV to AAA (Fig. 9*B*). The interaction between Csmd2 and PSD-95 was completely abolished when the PDZ-binding motif in Csmd2 was mutated (Fig. 9*C*). These data confirm that Csmd2 interacts with PSD-95 via a PDZ-binding domain at the C terminus of the cytoplasmic tail.

Based on our data showing colocalization of Csmd2 with PSD-95 at synapses, we hypothesized that the Csmd2 PDZ domain would be important for synaptic localization of Csmd2. To test this, we conducted *in utero* electroporation experiments to express wild-type FLAG-Csmd2 or the version in which the PDZ-binding domain is mutated (Fig. 9*D*). We electroporated the wild-type or mutant constructs into embryonic mouse cortices at E15.5 and conducted Percoll fractionations at P30, followed by Western blot analysis (Fig. 9*D*). Similar to endogenous Csmd2, FLAG-Csmd2 was found enriched in synaptosome-containing fractions 3 and 4 (Fig. 9*E*). Conversely, the PDZ-binding mutant version was primarily found in fractions 1 and 2 (Fig. 9*E*). Even when we increased sensitivity of the assay by employing a FLAG IP to enrich for the tagged protein, we could barely detect any mutant version in the synaptosomal fractions F3-F4 (Fig. 9*F*).

In a complementary approach, we transfected primary hippocampal neuron cultures with a FLAG-tagged PDZ-binding mutant of Csmd2 (Fig. 9*G*). In contrast to the wild-type protein (Fig. 6*A*), mutant Csmd2 no longer colocalized with PSD-95 in dendritic spines at 21 DIV (Fig. 9*G*). Although some faint FLAG signal was detected at the base of primary dendrites, the mutant protein was mostly restricted to the cell bodies of transfected neurons. Importantly, the wild-type and mutant Csmd2 constructs were equally expressed on the plasma membrane of HEK293T cells (Fig. 9*H*) While these samples were permeabilized, FLAG signal localization matched that of myr-tdTomato, indicating similar trafficking patterns. Together, these data indicate that the synaptic localization of Csmd2 depends on its intracellular PDZ-binding domain, possibly through its interactions with PDZ-containing synaptic scaffold proteins like PSD-95.

### Csmd2 is required for dendrite and dendritic spine development

**Figure 10.**
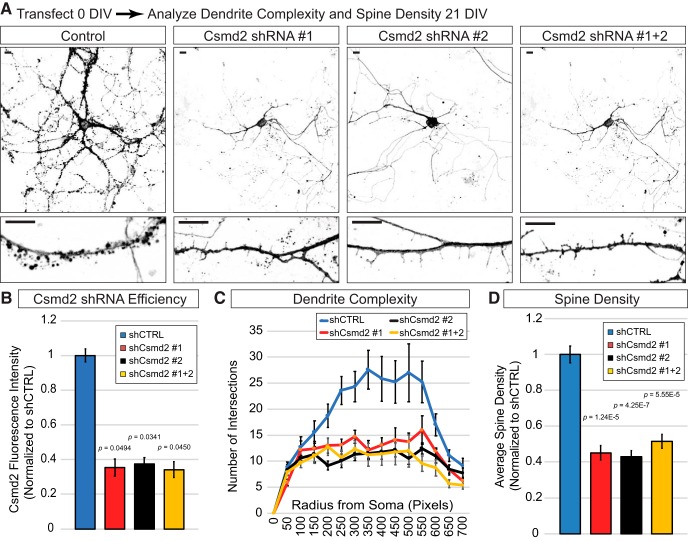
Csmd2 loss of function results in reduced dendritic spine density and dendrite complexity. ***A***, Dissociated neurons from E17.5 hippocampus were transfected at 0 DIV with non-targeting control or Csmd2 shRNA vectors that also express myristoylated-tdTomato as a transfection marker and to reveal cell morphology. Cells were transfected either with control shRNA, Csmd2 shRNA #1, Csmd2 shRNA #2, or both Csmd2 shRNAs together at half concentration each. Morphologic complexity and dendritic spine density were analyzed at 21 DIV. ***B***, Transfected cells were stained with α-Csmd2 (Novus) at three DIV to assess Csmd2 knock-down levels. Graph shows quantification (average ± SEM of biological replicates) of Csmd2 immunocytochemistry signal in transfected cells, relative to the non-targeting control shRNA. ***C***, ***D***, Quantification (average ± SEM of biological replicates) of dendrite complexity (***C***) and spine densities (***D***). For the dendrite complexity graph in ***C***, all shCsmd2 treatments exhibited statistical significance (*p* < 0.05) between 200 and 650 pixels from the soma. Scale bars, 10 μm.

Our data led us to next ask whether Csmd2 is required to form proper dendrites and synapses. We knocked down *Csmd2* mRNA in dissociated hippocampal neurons by the introduction of plasmids expressing shRNAs targeting *Csmd2* (Fig. 10). The shRNA plasmids used also contained a myr-tdTomato expression cassette to label the plasma membranes of transfected cells. Plasmids were transfected on the day the neurons were plated (0 DIV). We confirmed that each of the two shRNAs were capable of knocking down Csmd2 protein levels by >60% within 3 DIV (Fig. 10*B*). Furthermore, similar knock-down efficiency was achieved by combining half the amounts of each shRNA (Fig. 10*B*, shCsmd2 #1 + 2), thus allowing for greater specificity and fewer potential off-target effects. Next, we allowed the transfected neurons to develop to 21 DIV, at which point we analyzed dendrite complexity and dendritic spine density (Fig. 10*A*). Compared to neurons transfected with a non-targeting shRNA control construct, neurons transfected with *Csmd2*-targeting shRNA constructs displayed reduced dendritic complexity as measured by Sholl analysis (Fig. 10*A–C*). Additionally, *Csmd2* knock-down resulted in fewer dendritic spines in each treatment group (Fig. 10*A*,*D*).

**Figure 11.**
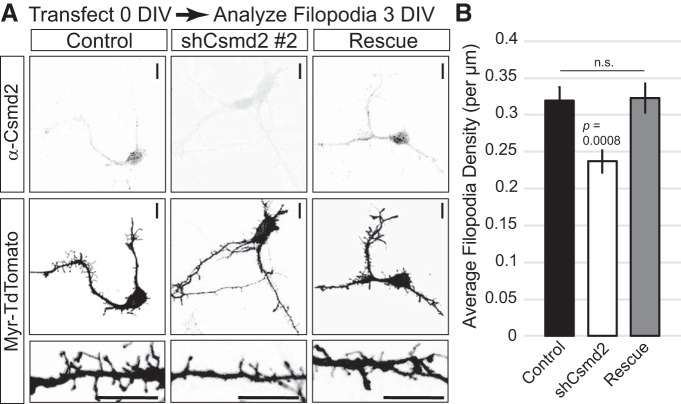
Csmd2 is required for development of neuronal filopodia in developing neurons. ***A***, shRNA-mediated knock-down of Csmd2 in hippocampal neurons (shRNA #2 targeting the 3’UTR) resulted in a 25% decrease in filopodia density as visualized by myr-tdTomato expression and quantified in ***B*** (average ± SEM of biological replicates). This deficit was rescued by the simultaneous expression of an shRNA-resistant construct for the expression of full-length Csmd2. Scale bars, 10 μm.

**Figure 12.**
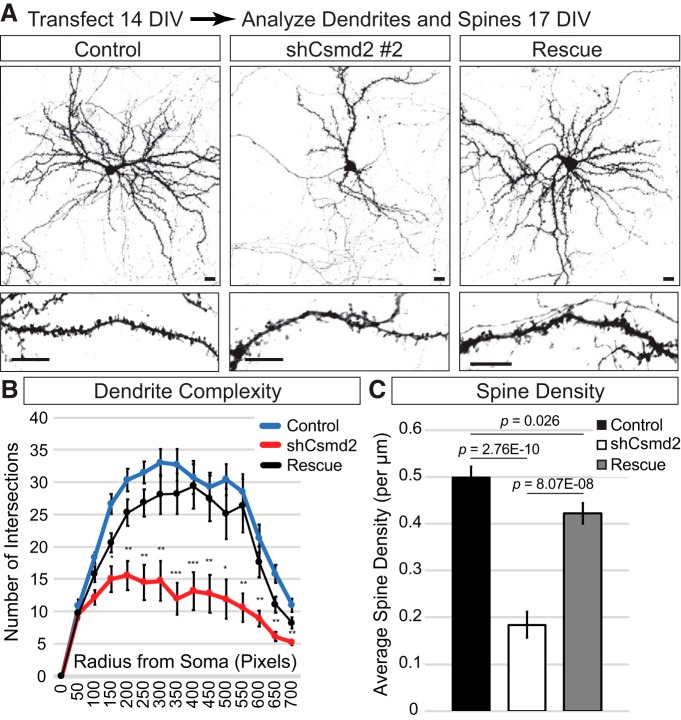
Csmd2 is required for maintenance of dendritic arbors and spines. ***A***, shRNA-mediated knock-down of Csmd2 at 14 DIV (shRNA #2 targeting the 3’UTR) caused reduced dendritic arbor complexity and decreased dendritic spine density at 17 DIV, compared to controls. Co-transfection of a refractory Csmd2 cDNA completely rescued dendritic arbor defects and partially restored spine density to control levels. ***B***, ***C***, Quantification (average ± SEM for biological replicates) of dendrite complexity (***B***) and dendritic spine density (***C***); *p < 0.05, **p < 0.01, ***p < 0.001; compared to non-targeting control shRNA group. Scale bars, 10 μm.

We next wanted to address whether Csmd2 was important for initial formation of dendritic spines, or their long-term maintenance. To test initial formation, we designed short-term experiments to evaluate the role of Csmd2 in filopodial development 3 d after transfection of dissociated hippocampal neurons at 0 DIV (Fig. 11). We observed a 25% reduction in filopodia density on *Csmd2* knock-down at 3 DIV (Fig. 11*A*,*B*). Re-introduction of a Csmd2 cDNA that is refractory to the shRNA used brought Csmd2 protein back to control levels and completely rescued filopodia density (Fig. 11*B*). To examine the role of Csmd2 in dendrite and dendritic spine maintenance, we knocked down *Csmd2* expression in neurons at 14 DIV and assessed dendritic spine density and dendrite complexity at 17 DIV (Fig. 12*A*). Sholl analysis revealed a significant reduction in dendrite complexity in Csmd2 knockdown neurons compared to controls (Figure 12*B*), which was rescued by restoration of Csmd2 levels. Similarly, shRNA-mediated knockdown of *Csmd2* expression resulted in an approximately 60% reduction in dendritic spine density, which was partially rescued by re-introduction of refractory Csmd2 (Figure 12*C*). We conclude that Csmd2 is required for the initial formation of dendritic filopodia, as well as the maintenance of dendritic spines and the more mature dendritic arbor.

## Discussion

Genetic variations in the human *CSMD* genes have been associated with the onset of schizophrenia and autism spectrum disorder in a number of GWAS studies, suggesting that alterations in the *CSMD* family contribute to neuropsychiatric disease (Havik et al., 2011; Donohoe et al., 2013; Steen et al., 2013; Koiliari et al., 2014; Sakamoto et al., 2016). However, the normal functions of CSMD proteins has remained largely unknown. Here, we show that mouse Csmd2 is expressed in the forebrain in multiple excitatory and inhibitory neuron types, where it localizes to dendrites and dendritic spines. We further identify synaptic scaffolding proteins, including PSD-95, as interactors with Csmd2. The interaction of Csmd2 with PSD-95 and its synaptic localization require a PDZ-binding domain in the Csmd2 cytoplasmic tail. Finally, we use Csmd2 loss-of-function experiments in dissociated hippocampal neurons to demonstrate that Csmd2 is required for the formation and maintenance of dendritic spines and the dendritic arbor. Taken together, these data indicate that Csmd2 is a novel synaptic transmembrane protein and ultimately point toward a synaptic function for this previously uncharacterized protein.

The molecular mechanisms by which Csmd2 regulates dendrite and synapse formation remain to be elucidated, but we may gain some insights from the roles of other CUB and/or Sushi domain containing proteins. For example, CUB/Sushi-containing proteins such as Lev9/10 and Neto1/2 play significant roles as auxiliary subunits of synaptic receptors. Specifically, Neto1 and Neto2 are responsible for phosphorylation-dependent regulation of kainate receptor subunit composition (Fisher and Mott, 2013; Lomash et al., 2017; Wyeth et al., 2017). Neto1 maintains the synaptic localization of NR2A subunit-containing NMDA receptors (NMDARs) and thus mediate long-term potentiation (LTP; Ng et al., 2009; Cousins et al., 2013). Additionally, Lev9 and Lev10 proteins are responsible for acetylcholine receptor clustering at the neuromuscular junction, thus also regulating synapse composition and function (Gendrel et al., 2009). Future work will pursue the question of whether Csmd2 functions similarly with iGluRs at the synapse and thus regulate synapse function. In this context, it will be important to identify the extracellular binding partners of Csmd2, and whether Csmd2 may mediate their trafficking, clustering and functions at excitatory synapses. Interestingly, the closely related protein Csmd1 was recently identified in a proteomic screen for inhibitory synaptic cleft proteins (Loh et al., 2016), and we find that the highest expression of Csmd2 in the forebrain is in PV^+^ and SST^+^ inhibitory interneurons. Future work will pursue the cellular and physiologic functions of Csmd2 in GABAergic interneurons in the neocortex, which may provide deeper insight into a role for Csmd2 in maintaining the correct balance of excitatory/inhibitory connectivity in forebrain neural circuits.

Our data also demonstrate a requirement for Csmd2 in dendrite arborization, similar to a recently reported role for Csmd3 in dendrite development (Mizukami et al., 2016). Given that synaptic activity is widely understood to play a significant role in dendrite development and remodeling, it will be interesting to characterize changes in synapse composition and activity on Csmd2 loss-of-function. This would point to a potential activity-dependent function of Csmd2 that, in turn, mediates the development and remodeling of the dendritic arbor.

In conclusion, we have characterized the subcellular localization and function Csmd2, a protein of previously unknown function, in the context of dendrite and dendritic spine development. Future studies focusing on the function of this protein in the central nervous system may lead to a clearer understanding of the molecular mechanisms governing dendrite and synapse formation and function. Such studies may provide a new insight into the underlying causes of psychiatric disorders associated with defects in neural circuit connectivity, such as schizophrenia and autism spectrum disorder.

## Synthesis

Reviewing Editor: Esther Stoeckli, University of Zurich Decisions are customarily a result of the Reviewing Editor and the peer reviewers coming together and discussing their recommendations until a consensus is reached. When revisions are invited, a fact-based synthesis statement explaining their decision and outlining what is needed to prepare a revision will be listed below.

We agree that the study characterizing the localization of Csmd2 and its role in neuronal maturation is interesting and that it contributes to our understanding of these large proteins. The link of Csmd proteins to neuropsychiatric disorders prompt their functional characterization during neural development. The authors use immunohistochemistry and in situ hybridization to localize the protein or mRNA, respectively. They localize Csmd2 not only to the somato-dendritic compartment but also to the post-synaptic density. In dendritic spines of excitatory neurons smd2 co-localizes with PSD-95. Functional perturbation with transfection of shRNA, Csmd2 is shown to affect filopodia/spine number and dendritic complexity.

However, we all agreed that some essential control experiments are missing. Our major concern was the specificity of the shRNA that was used. The specificity and the efficiency of the shRNA needs to be tested. Ideally, more than one shRNA should be used. The sequence of the shRNA and the rescue construct needs to be described in detail.

Similarly, we found that the specificity of the antibodies needs to be tested more rigorously. In the absence of Csmd2 knockout tissue, the specificity could be shown in shRNA-treated neurons. Please find more detailed suggestions in the reviewers' comments that I add below.

Major Points

1. No validation is provided for the shRNA against Csmd2 used in Figures 9-11. How specific is this shRNA and how effective is it in reducing Csmd2 expression, which is a very large protein? Specificity can be demonstrated in e.g. HEK293 cells expressing Csmd1, 2, 3. Efficiency can be demonstrated in HEK293 cells as well, but better would be to demonstrate knockdown of the endogenous protein in cell lysates of cultured neurons expressing shCsmd2. It appears that only one shRNA was used. What is the design (e.g. what was the sequence) for this?

Even if the rescues seem to work nicely, it is possible that the co-transfection itself rendered the KD constructs inefficient. Along these lines, it is also possible that the KD constructs worked non-specifically, which is often a concern using shRNAs. Showing qPCR of westerns demonstrating efficacy of the KD would be very helpful here. Another standard control might be to include a scrambled shRNA control and a different shRNA targeting Csmd2.

2. Antibody-staining and immuno-localization:

* In one hand the Authors highlight that Csmd2 is localized somatically (Figs 2&3), on the other hand they also argue that its localization in spines is important (Fig 4). But it is not clear why the first was evaluated using the “Novus” antibody, the second was evaluated using a different antibody, the “SC-G19”.

* Based on Fig2D, I find it hard to have confidence in the specificity of the Novus antibody. The left panel (Novus) and the right panel (No Primary Ab) seem to show very similar structures, with a large difference in the background. In Fig2E lower panels (No Primary Ab), the background is less concerning. However, here it is puzzling why the CA1 and DG cell bodies are not visible, when Fig3 argues for somatic expression of Csmd2 in these cells. Validating these antibodies in Csmd2 KO mice would be a valuable addition, if these mice are available. If not, the ms should at least discuss this issue more upfront.

* Fig2 evaluates the presence of Csmd2 in projection neurons and in interneurons using only 1-2 single cells as examples for each. To support the conclusions, it would be advantageous to present larger cell numbers. Especially in the case of astrocytes (Aldh1l1), which should be abundantly present.

3) Yeast 2-hybrid screen: this is an integral part of the study, and subsequent PSD-95 binding experiments validate the approach. However, the Authors need to include more details about their methods and results. What were the blank and binding values? How was the confidence determined? Unfortunately, the methods section do not provide much information on this either.

Minor Points

1. Figure 2D, E: In the absence of Csmd2 knockout brain tissue, it would be more convincing if the authors could show an antibody absorption control to demonstrate specificity of the immunohistochemical signal. In addition, they could test their antibody on Csdm2 shRNA-expressing neurons to demonstrate specificity.

2. Figure 2E contains diagonal blue stripes, likely an artefact of sectioning. This image should be replaced or simply removed.

3. The image in Figure 5A is not convincing: the membrane of the cultured DIV14 hippocampal neuron looks weird, possible due to high levels of expression of myr-tdTomato in this cell. In addition, the Csmd2 puncta are very small and ubiquitous. This distribution of Csmd2 looks quite different from the one shown in electroporated tissue (Figure 4). Panel 5A should be removed. It would be interesting to show the distribution of FLAG-CSMD2 in cultured neurons instead.

4. Figure 8: this is a nice experiment but see previous minor point 3; it would be good to validate the distribution of FLAG-Csmd2 constructs: are both targeted to the plasma membrane? Does Csmd2 WT target to spines and is this altered for the PDZ-mut construct?

5. Loh et al Cell 2016 showed localization of Csmd1 to the synapse using proximity biotinylation of synaptic cleft proteins - this paper supports the findings of the authors and should be cited.

6. Page 11 small typo: ‘syntaptosomal’.

## Author Response

We would like to thank the reviewers for their thoughtful and constructive comments, which have helped us to improve the manuscript. The reviewers recognized and appreciated the novelties and significance of the research, and they raised additional points and valid criticisms. We have carefully revised the manuscript in accordance with the synthesized comments, addressing their concerns with additional data and revised text.

The overall revisions that are now included are summarized here:

• We focused a significant amount of our efforts on essential control experiments validating the shRNA knockdown strategy and Csmd2 antibodies. o shRNA {section sign} We have now incorporated a non-targeting control shRNA as well as an additional shRNA targeting Csmd2. {section sign} We also included experiments in which the two different Csmd2 shRNAs are used together at half of their normal concentrations to further address specificity concerns. {section sign} We added new data to revised Figure 10 in which we quantify the efficiency of Csmd2 knockdown with each shRNA and the combination compared to non-targeting control, using immunocytochemistry on transfected primary neuronal cultures. {section sign} We have added sequence details of each shRNA construct to the revised methods section. o Antibodies {section sign} We show that 3 different antibodies similarly recognize Csmd2 by Western blot and immunocytochemistry when Csmd2 is heterologously expressed in HEK293T cells (revised Figure 2). {section sign} We show overviews and cellular detail that two of the Csmd2 antibodies give identical staining patterns by immunohistochemistry on brain sections (revised Figure 3). IHC with secondary antibodies alone show no staining. {section sign} We show by immunocytochemistry on primary neuronal cultures that Csmd2 signal is decreased upon expression of shRNAs targeting Csmd2 (revised Figures 10, 11 and 12).

Below is our point-by-point response to the synthesized reviewers' comments:

• “No validation is provided for the shRNA against Csmd2 used in Figures 9-11.” o We now provide quantification in revised Figure 10 on the efficiency of Csmd2 knockdown by each shRNA. o We have now included a control shRNA that does not match any known mammalian mRNAs. o We also include a standard test of specificity/off-target effects by combining two different shRNAs at half the normal concentration. Off-target effects are expected to be different for each shRNA and minimized by using the half dose, so any combined action of the two low-dose shRNAs is much more likely to be from their specific effects on Csmd2 mRNA. {section sign} We do not have antibodies against Csmd1 or 3 and have not yet been able to clone either cDNA, so we could not test the shRNAs against these two family members. However, the shRNAs were designed to regions that do not share significant homology with either of these mRNAs. • “It appears that only one shRNA was used. What is the design (e.g. what was the sequence) for this?”

o We have now included much more detail of the sequences used and a better description of the experimental design in the revised methods section. o We originally used the combination of the two shRNAs in our first submission, but failed to describe this adequately. We now validate the individual shRNAs alone and show they recapitulate the phenotype seen with the combination (revised Figure 10). • “In one hand the Authors highlight that Csmd2 is localized somatically (Figs 2&3), on the other hand they also argue that its localization in spines is important (Fig 4). But it is not clear why the first was evaluated using the “Novus” antibody, the second was evaluated using a different antibody, the “SC-G19”.” o We have added new data and revised the figures and text/methods to be more clear about this point. We do see the same staining pattern - soma + dendrites + spines - using both the Novus antibody and the SC-G19 antibody. We now show results from both antibodies in revised Figure 3. {section sign} However, the SC-G19 antibody works much more consistently for IHC than the Novus antibody, which can often give no signal at all if the tissue processing is not optimal. Therefore we use the SC-G19 antibody to demonstrate which cell types express Csmd2 in revised Figure 4. {section sign} Unfortunately, the SC-G19 antibody was made and sold by Santa Cruz Biotech and they no longer sell this antibody. Since the Novus antibody works better by ICC than it does by IHC and our supply of SC-G19 antibody is now very limited, we performed all of our ICC using the Novus antibody. • “Based on Fig2D, I find it hard to have confidence in the specificity of the Novus antibody. The left panel (Novus) and the right panel (No Primary Ab) seem to show very similar structures, with a large difference in the background.” o Displaying the precise staining pattern of Csmd2 by IHC can be challenging due to the fact that it is highest in the soma and proximal dendrite, but is also expressed throughout the dendritic neuropil. We agree that the original images were not sufficient to display this accurately. {section sign} We have now added images showing the cellular detail of the somatodendritic and punctate neuropil staining in revised Figure 3. We think these new images more obviously show all of the different cellular expression domains for Csmd2, which are not present at all in the no-primary controls. {section sign} We would like to note that although some of the staining in the overview images looks like background staining at low magnification, the higher magnification images more clearly show that these are discrete structures within the dendritic neuropil. • “it is puzzling why the CA1 and DG cell bodies are not visible, when Fig3 argues for somatic expression of Csmd2 in these cells.” o This is a good point and we have now provided more clear higher mag images of the CA1 cell bodies that are positive for Csmd2. • “Fig2 evaluates the presence of Csmd2 in projection neurons and in interneurons using only 1-2 single cells as examples for each. To support the conclusions, it would be advantageous to present larger cell numbers. Especially in the case of astrocytes (Aldh1l1), which should be abundantly present.” o We have now revised this figure (now Figure 4) to include multiple examples of each cell neuronal cell type. o We have been unable to obtain consistent Csdm2 staining patterns in glial cells. Csmd2 signal is always weaker in these cell types when present, but often is undetectable at all. Because of this, we have removed the astrocyte (Aldh1l1) and

oligodendrocyte (Olig2) stainings and now mention in the text that our results were inconclusive on whether Csmd2 is expressed in glial cells. • “Yeast 2-hybrid screen: the Authors need to include more details about their methods and results.” o Thank you for pointing out this oversight. We now include more detail on the Y2H screen methods and results. {section sign} We also now include an Extended Data set with the entire Y2H screen results.

• MINOR POINTS o “Figure 2D, E: In the absence of Csmd2 knockout brain tissue, it would be more convincing if the authors could show an antibody absorption control to demonstrate specificity of the immunohistochemical signal. In addition, they could test their antibody on Csdm2 shRNA-expressing neurons to demonstrate specificity.” {section sign} We do now provide evidence that the Csmd2 staining is decreased upon shRNA-mediated knockdown of Csmd2 (revised Figures 10-12). {section sign} Unfortunately we do not know the antigen used to generate the SC-G19 antibody to test an antibody absorption control. This information was not provided by the supplier upon our request. o “Figure 2E contains diagonal blue stripes, likely an artefact of sectioning. This image should be replaced or simply removed.” {section sign} This has been removed in the revised Figure 3. o “Panel 5A should be removed. It would be interesting to show the distribution of FLAG-CSMD2 in cultured neurons instead.” {section sign} We followed this excellent suggestion and removed this panel and replaced it with the distribution of FLAG-Csmd2 in cultured neurons in revised Figure 6. o “Figure 8: this is a nice experiment but see previous minor point 3; it would be good to validate the distribution of FLAG-Csmd2 constructs: are both targeted to the plasma membrane?” {section sign} We now show in revised Figure 9 that both proteins correctly target to the plasma membrane in HEK293T cells. o “Does Csmd2 WT target to spines and is this altered for the PDZ-mut construct?” {section sign} We now show in revised Figures 6 and 9 that the WT protein targets to spines but the PDZ-mut does not. o “Loh et al Cell 2016 showed localization of Csmd1 to the synapse using proximity biotinylation of synaptic cleft proteins - this paper supports the findings of the authors and should be cited.” {section sign} Thank you for bringing this reference to our attention - we now include a discussion of this paper in our text. o “Page 11 small typo: ‘syntaptosomal’.” {section sign} This has been corrected.

Thank you again for all the helpful suggestions and comments. We look forward to hearing from you soon regarding our submission. We would be glad to respond to any further questions and comments that you may have
