# Supplementary material for: Csmd2 Is a Synaptic Transmembrane Protein that Interacts with PSD-95 and Is Required for Neuronal Maturation
Source: eNeuro. 2019 May 7;6(2):ENEURO.0434-18.2019. doi: 10.1523/ENEURO.0434-18.2019 (PMC6506821; doi:10.1523/ENEURO.0434-18.2019)
Supplement: Extended Data Figure 8-1 — Complete Yeast 2-Hybrid Results Summary. For each interaction, a Predicted Biological Score (PBS) is computed to assess the interaction reliability. This score represents the probability of an interaction to be non-specific: it is an e-value, primarily based on the comparison between the number of independent prey fragments found for an interaction and the chance of finding them at random (background noise). The value varies between 0 and 1. Several thresholds have been arbitrarily defined in order to rank the results in 4 categories from A (the highest confidence rank) to D. PBS D generally represents interactions identified through one unique prey fragment or multiple identical ones. It can be interactions hardly detectable by the Y2H technique (low representation of the mRNA in the library, prey folding, prey toxicity in yeast) or it can be false-positive interactions. The PBS is adjusted by integrating the PBS of other interactions from the database in which interaction domains of the involved proteins have been found. For example, reciprocal interactions found in independent screens are technically very reliable and thus tagged as A, B or C. Two additional categories have been implemented: PBS E and PBS F. The PBS E represents interactions involving prey domains connected to more than 10 different mouse bait proteins in the entire database. This arbitrary threshold allows flagging of highly – or relatively highly – connected protein domains. Experimentally proven artifacts of the Y2H technology are flagged with a PBS F. These can be LexA or Gal4 protein binders or binders of the DNA sequence upstream of the reporter gene. Figure 8-1, PDF file. [file sup_enu-eN-NWR-0434-18-s01.pdf]

Figure 8-1. Complete Yeast 2-Hybrid Results Summary. For each interaction, a Predicted Biological Score (PBS) is computed to assess the interaction reliability. This score represents the probability of an interaction to be non-specific: it is an e-value, primarily based on the comparison between the number of independent prey fragments found for an interaction and the chance of finding them at random (background noise). The value varies between 0 and 1. Several thresholds have been arbitrarily defined in order to rank the results in 4 categories from A (the highest confidence rank) to D. PBS D generally represents interactions identified through one unique prey fragment or multiple identical ones. It can be interactions hardly detectable by the Y2H technique (low representation of the mRNA in the library, prey folding, prey toxicity in yeast) or it can be false-positive interactions. The PBS is adjusted by integrating the PBS of other interactions from the database in which interaction domains of the involved proteins have been found. For example, reciprocal interactions found in independent screens are technically very reliable and thus tagged as A, B or C. Two additional categories have been implemented: PBS E and PBS F. The PBS E represents interactions involving prey domains connected to more than 10 different mouse bait proteins in the entire database. This arbitrary threshold allows flagging of highly – or relatively highly – connected protein domains. Experimentally proven artifacts of the Y2H technology are flagged with a PBS F. These can be LexA or Gal4 protein binders or binders of the DNA sequence upstream of the reporter gene.

HYBRIGENICS SERVICES

Results Summary

ULTImate Y2H SCREEN
Mus musculus - Csmd2
# vs Mouse Adult Brain_RP1

Wed, Dec 24, 2014 - 04:02 PM

Screen Parameters

Nature	cDNA
Reference Bait Fragment	Mus musculus - Csmd2 (aa 3557-3611) ; hgx3854v1
Prey Library	Mouse Adult Brain_RP1
Vector(s)	pB27 (N-LexA-bait-C fusion)
Processed Clones	177 (pB27_A)
Analyzed Interactions	88.7 millions (pB27_A)
3AT Concentration	0.5 mM (pB27_A)

Global PBS®

Global PBS (for Interactions represented in the Screen)		Nb	%
A	Very high confidence in the interaction	5	29.4%
B	High confidence in the interaction	6	35.3%
C	Good confidence in the interaction	2	11.8%
D	Moderate confidence in the interaction<br>This category is the most difficult to interpret because it mixes two classes of interactions :<br>- False-positive interactions<br>- Interactions hardly detectable by the Y2H technique (low representation of the mRNA in the library, prey folding, prey toxicity in yeast)	2	11.8%
E	Interactions involving highly connected (or relatively highly connected) prey domains, warning of non-specific interaction. The total number of screens performed on each organism is taken into account to set this connectivity threshold: 20 interactions to different bait proteins in our entire database for Human, 10 for Mouse, Drosophila and Arabidopsis and 6 for all other organisms. They can be classified in different categories:<br>- Prey proteins that are known to be highly connected due to their biological function<br>- Proteins with a prey interacting domain that contains a known protein interaction motif or a biochemically promiscuous motif	2	11.8%
F	Experimentally proven technical artifacts	0	0.0%
**Non Appliable**			
N/A	The PBS is a score that is automatically computed through algorithms and cannot be attributed for the following reasons :<br>- All the fragments of the same reference CDS are antisense<br>- The 5p sequence is missing<br>- All the fragments of the same reference CDS are either all OOF1 or all OOF2<br>- All the fragments of the same reference CDS lie in the 5' or 3' UTR		

HYBRIGENICS CONFIDENTIAL - 949116 - S. Franco

Prey Fragment Analysis

Symbols	Means
	The fragment contains the full length CDS
	Fragment is fully in 5' UTR
	Fragment is fully in 3' UTR
	Fragment contains at least one In Frame STOP codon
[NR]	Fragment was found to be non relevant (poor quality, high N density)
IF<br>OOF1<br>OOF2	With regard to the theoretical frame of each corresponding CDS (GeneBank),fragments are cloned in frame (IF) if they are in the same frame as Gal4AD.In general, polypeptides synthesized from OOF fragments are not considered of biological interest, unless found together with another frame. However, some of the proteins expressed from an OOF fragment can be translated in the correct frame,due to the existence of natural frame-shift events during translation in yeast
??	Unidentified frame when :<br>- The clone sequence is antisense<br>- The 5p sequence is missing
N	Antisense
Start...Stop	Position of the 5p and 3p prey fragment ends, relative to the position of the ATG start codon (A=0)

Clone Name	Type Seq	Gene Name (Best Match)	Start..Stop (nt)	Frame	Sense	%Id 5p	%Id 3p	PBS
pB27_A-21	3p	Mus musculus - Dlg1 var 2	..1984	??			94.1	A
pB27_A-80	5p/3p	Mus musculus - Dlg1 var 2	111..1541	IF		96.6	93.7	A
pB27_A-166	5p/3p	Mus musculus - Dlg1 var 2	400..1546	OOF1		90.7	93.5	A
pB27_A-37	5p/3p	Mus musculus - Dlg1 var 2	459..1436	IF		93.8	94.2	A
pB27_A-16	5p/3p	Mus musculus - Dlg1 var 2	459..1436	IF		95.6	93.5	A
pB27_A-185	5p/3p	Mus musculus - Dlg1 var 2	483..1421	IF		96.9	93.6	A
pB27_A-143	5p/3p	Mus musculus - Dlg1 var 2	588..1505	IF		93.7	95.4	A
pB27_A-167	5p/3p	Mus musculus - Dlg1 var 2	591..1443	IF		93.8	91.6	A
pB27_A-130	5p/3p	Mus musculus - Dlg1 var 2	591..1443	IF		84.6	80.9	A
pB27_A-123	5p/3p	Mus musculus - Dlg1 var 2	591..1443	IF		94.8	95.7	A
pB27_A-70	5p/3p	Mus musculus - Dlg1 var 2	591..1443	IF		96.0	97.0	A
pB27_A-75	5p/3p	Mus musculus - Dlg1 var 2	591..1443	IF		96.1	97.9	A
pB27_A-148	5p/3p	Mus musculus - Dlg1 var 2	597..1541	IF		96.5	94.1	A
pB27_A-84	5p/3p	Mus musculus - Dlg1 var 2	597..1541	IF		96.5	91.9	A
pB27_A-85	5p/3p	Mus musculus - Dlg1 var 2	597..1541	IF		95.8	93.7	A
pB27_A-25	5p/3p	Mus musculus - Dlg1 var 2	597..1541	IF		91.5	92.0	A
pB27_A-78	5p/3p	Mus musculus - Dlg1 var 2	609..1532	IF		92.8	96.3	A
pB27_A-14	5p/3p	Mus musculus - Dlg1 var 2	609..2366	IF		95.9	85.1	A
pB27_A-76	5p/3p	Mus musculus - Dlg1 var 2	609..1495	IF		96.5	96.7	A
pB27_A-8	5p/3p	Mus musculus - Dlg1 var 2	627..1663	IF		95.4	89.2	A
pB27_A-83	5p/3p	Mus musculus - Dlg1 var 2	639..1543	IF		95.2	94.1	A
pB27_A-56	5p/3p	Mus musculus - Dlg1 var 2	639..1543	IF		94.4	93.4	A
pB27_A-48	5p/3p	Mus musculus - Dlg1 var 2	642..1324	IF		98.3	97.3	A
pB27_A-72	5p/3p	Mus musculus - Dlg1 var 2	642..1324	IF		99.0	89.2	A
pB27_A-35	5p/3p	Mus musculus - Dlg1 var 2	660..1798	IF		90.8	85.4	A
pB27_A-135	5p/3p	Mus musculus - Dlg1 var 2	708..1445	IF		95.2	90.8	A
pB27_A-171	5p/3p	Mus musculus - Dlg1 var 2	714..2104	IF		95.4	84.2	A
pB27_A-133	5p/3p	Mus musculus - Dlg1 var 2	720..1494	IF		95.5	94.4	A
pB27_A-90	5p/3p	Mus musculus - Dlg1 var 2	735..2496	IF		95.7	83.5	A

Clone Name	Type Seq	Gene Name (Best Match)	Start..Stop (nt)	Frame	Sense	%Id 5p	%Id 3p	PBS
pB27_A-50	5p/3p	Mus musculus - Dlg1 var 2	738..1960	IF		95.6	91.5	A
pB27_A-172	5p/3p	Mus musculus - Dlg1 var 2	741..1374	IF		99.4	99.5	A
pB27_A-186	5p/3p	Mus musculus - Dlg1 var 2	744..1798	IF		94.4	92.4	A
pB27_A-98	5p/3p	Mus musculus - Dlg1 var 2	744..1892	IF		88.8	88.8	A
pB27_A-92	5p/3p	Mus musculus - Dlg1 var 2	747..1680	IF		96.2	95.9	A
pB27_A-174	5p	Mus musculus - Dlg1 var 2	753	IF		96.8		A
pB27_A-43	5p/3p	Mus musculus - Dlg1 var 2	762..1602	IF		94.1	85.4	A
pB27_A-99	5p/3p	Mus musculus - Dlg1 var 2	768..1426	IF		90.5	85.7	A
pB27_A-113	5p/3p	Mus musculus - Dlg1 var 2	789..1437	IF		88.4	89.7	A
pB27_A-88	5p/3p	Mus musculus - Dlg1 var 2	789..1437	IF		98.9	97.9	A
pB27_A-39	5p/3p	Mus musculus - Dlg1 var 2	789..1936	IF		96.5	95.1	A
pB27_A-62	5p/3p	Mus musculus - Dlg1 var 2	789..1437	IF		99.2	97.2	A
pB27_A-145	5p/3p	Mus musculus - Dlg1 var 2	792..1505	IF		98.5	94.1	A
pB27_A-66	5p/3p	Mus musculus - Dlg1 var 2	798..1987	IF		97.0	93.4	A
pB27_A-158	5p/3p	Mus musculus - Dlg1 var 2	810..1990	IF		87.7	89.7	A
pB27_A-132	5p/3p	Mus musculus - Dlg1 var 2	810..1438	IF		99.6	94.5	A
pB27_A-86	5p/3p	Mus musculus - Dlg1 var 2	915..2042	IF		94.4	91.3	A
pB27_A-106	5p/3p	Mus musculus - Dlg1 var 2	939..1900	IF		98.9	90.1	A
pB27_A-120	3p	Mus musculus - Dlg1 var 3	..2456	??			88.8	N/A
pB27_A-164	3p	Mus musculus - Dlg1 var 3	..2303	??			89.1	N/A
pB27_A-59	3p	Mus musculus - Dlg1 var 3	..2456	??			91.6	N/A
pB27_A-79	3p	Mus musculus - Dlg1 var 3	..2303	??			90.9	N/A
pB27_A-107	3p	Mus musculus - Dlg1 var 5	..1396	??			91.8	A
pB27_A-181	3p	Mus musculus - Dlg1 var 5	..1742	??			92.9	A
pB27_A-187	3p	Mus musculus - Dlg1 var 5	..1344	??			87.5	A
pB27_A-112	3p	Mus musculus - Dlg1 var 5	..1425	??			94.4	A
pB27_A-124	3p	Mus musculus - Dlg1 var 5	..1271	??			95.9	A
pB27_A-44	5p/3p	Mus musculus - Dlg1 var 5	573..1810	IF		91.1	93.6	A
pB27_A-115	5p/3p	Mus musculus - Dlg1 var 5	573..1810	IF		89.6	92.2	A
pB27_A-153	5p	Mus musculus - Dlg1 var 5	957	IF		97.5		A
pB27_A-89	5p	Mus musculus - Dlg1 var 5	957	IF		99.3		A
pB27_A-4	5p/3p	Mus musculus - Dlg1 var 5	1029..2320	IF		92.9	90.1	A
pB27_A-170	5p/3p	Mus musculus - Dlg1 var 5	1035..1907	IF		94.1	95.0	A
pB27_A-190	5p/3p	Mus musculus - Dlg1 var 5	1035..1907	IF		89.2	96.5	A
pB27_A-55	5p/3p	Mus musculus - Dlg1 var 5	1125..1890	IF		97.2	94.0	A
pB27_A-96	5p/3p	Mus musculus - Dlg2	-22..889	IF		93.2	92.4	A
pB27_A-17	5p/3p	Mus musculus - Dlg2	-22..889	IF		97.2	94.1	A
pB27_A-138	5p/3p	Mus musculus - Dlg2	-22..889	IF		91.0	83.8	A
pB27_A-110	5p/3p	Mus musculus - Dlg2	-22..889	IF		93.4	94.1	A
pB27_A-100	5p/3p	Mus musculus - Dlg2	84..727	IF		97.9	97.5	A
pB27_A-68	5p/3p	Mus musculus - Dlg2	89..985	OOF2		94.1	87.2	A
pB27_A-67	5p/3p	Mus musculus - Dlg2	89..985	OOF2		96.5	95.2	A
pB27_A-180	5p/3p	Mus musculus - Dlg2	89..985	OOF2		89.3	93.9	A
pB27_A-165	5p/3p	Mus musculus - Dlg2	189..763	IF		98.8	98.6	A
pB27_A-152	5p/3p	Mus musculus - Dlg2	327..1171	IF		86.9	90.1	A
pB27_A-128	5p/3p	Mus musculus - Dlg2	327..1171	IF		95.4	95.1	A
pB27_A-118	5p/3p	Mus musculus - Dlg2	357..993	IF		97.5	92.0	A
pB27_A-146	5p/3p	Mus musculus - Dlg2	360..1116	IF		93.4	94.9	A
pB27_A-29	5p/3p	Mus musculus - Dlg2	363..1342	IF		96.5	91.4	A
pB27_A-7	5p/3p	Mus musculus - Dlg2	363..820	IF		100.0	99.3	A
pB27_A-159	5p/3p	Mus musculus - Dlg2	453..1134	IF		98.1	96.1	A

Clone Name	Type Seq	Gene Name (Best Match)	Start..Stop (nt)	Frame	Sense	%Id 5p	%Id 3p	PBS
pB27_A-102	5p/3p	Mus musculus - Dlg2	519..1126	IF		98.4	90.8	A
pB27_A-19	5p/3p	Mus musculus - Dlg2	519..1126	IF		95.7	84.5	A
pB27_A-141	5p/3p	Mus musculus - Dlg2	1089..1810	IF		97.1	94.5	A
pB27_A-22	3p	Mus musculus - Dlg2	1257..1759	IF			100.0	A
pB27_A-49	5p/3p	Mus musculus - Dlg3	309..1204	IF		94.6	93.0	C
pB27_A-81	5p/3p	Mus musculus - Dlg3	324..1282	IF		96.6	94.2	C
pB27_A-73	3p	Mus musculus - Dlg4	..868	??			95.3	A
pB27_A-63	5p/3p	Mus musculus - Dlg4	31..772	OOF1		96.5	91.2	A
pB27_A-30	5p/3p	Mus musculus - Dlg4	31..772	OOF1		96.6	92.8	A
pB27_A-177	5p/3p	Mus musculus - Dlg4	57..860	OOF1		99.8	95.8	A
pB27_A-139	5p/3p	Mus musculus - Dlg4	57..860	OOF1		100.0	93.6	A
pB27_A-74	5p/3p	Mus musculus - Dlg4	57..860	OOF1		100.0	95.4	A
pB27_A-13	5p/3p	Mus musculus - Dlg4	58..860	OOF1		99.8	94.9	A
pB27_A-162	5p/3p	Mus musculus - Dlg4	70..1225	OOF1		95.6	95.8	A
pB27_A-150	5p/3p	Mus musculus - Dlg4	70..1225	OOF1		92.4	94.5	A
pB27_A-31	5p/3p	Mus musculus - Dlg4	70..1225	OOF1		94.2	94.1	A
pB27_A-137	5p/3p	Mus musculus - Dlg4	231..916	IF		92.8	81.6	A
pB27_A-23	5p/3p	Mus musculus - Dlg4	231..916	IF		98.1	96.9	A
pB27_A-20	5p/3p	Mus musculus - Dlg4	231..916	IF		96.1	95.6	A
pB27_A-95	5p/3p	Mus musculus - Dlg4	249..772	IF		99.8	99.8	A
pB27_A-38	5p/3p	Mus musculus - Dlg4	249..887	IF		99.4	95.0	A
pB27_A-47	5p/3p	Mus musculus - Dlg4	249..947	IF		98.3	94.0	A
pB27_A-27	5p/3p	Mus musculus - Dlg4	249..887	IF		94.7	92.1	A
pB27_A-2	5p/3p	Mus musculus - Dlg4	255..855	IF		99.8	96.3	A
pB27_A-155	5p/3p	Mus musculus - Dlg4	288..916	IF		97.5	94.8	A
pB27_A-11	5p/3p	Mus musculus - Dlg4	288..916	IF		89.7	93.4	A
pB27_A-131	5p/3p	Mus musculus - Dlg4	291..988	IF		95.8	98.6	A
pB27_A-1	5p/3p	Mus musculus - Dlg4	342..868	IF		99.6	90.9	A
pB27_A-53	5p/3p	Mus musculus - Dlg4	342..868	IF		99.6	99.6	A
pB27_A-168	5p/3p	Mus musculus - Dlg4	342..868	IF		92.9	86.2	A
pB27_A-126	5p/3p	Mus musculus - Dlg4	345..893	IF		90.3	99.3	A
pB27_A-65	5p/3p	Mus musculus - Dlg4	780..1412	IF		99.7	97.8	A
pB27_A-160	5p/3p	Mus musculus - Hmg20a	126..951	IF		95.1	94.4	E
pB27_A-173	5p/3p	Mus musculus - Hmg20a	126..951	IF		95.6	93.2	E
pB27_A-58	5p/3p	Mus musculus - Inadl	4068..5246	IF		97.2	95.8	B
pB27_A-105	5p/3p	Mus musculus - Inadl	4806..5448	IF		96.9	95.7	B
pB27_A-178	5p/3p	Mus musculus - Inadl	5022..5492	IF		100.0	99.8	B
pB27_A-117	5p/3p	Mus musculus - Inadl	5022..5492	IF		100.0	100.0	B
pB27_A-189	5p/3p	Mus musculus - Inadl	5085..6057 ✖	IF		93.0	93.8	B
pB27_A-61	5p/3p	Mus musculus - Magi1	2070..2880	IF		92.4	93.8	D
pB27_A-10	5p	Mus musculus - Magi2	429	IF		94.3		B
pB27_A-3	5p/3p	Mus musculus - Magi2	2198..3122	OOF2		90.2	91.4	B
pB27_A-184	5p/3p	Mus musculus - Magi2	2712..3119	IF		99.8	100.0	B
pB27_A-54	3p	Mus musculus - Magi3	..2482	??			77.7	B
pB27_A-94	5p/3p	Mus musculus - Magi3	754..2081	OOF1		95.2	93.5	B
pB27_A-87	5p/3p	Mus musculus - Magi3	1305..2290	IF		91.0	93.8	B
pB27_A-149	5p/3p	Mus musculus - Magi3	1305..2290	IF		95.6	97.7	B
pB27_A-169	5p/3p	Mus musculus - Magi3	1323..2417	IF		94.7	92.8	B
pB27_A-154	3p	Mus musculus - Mast1	..2739	??	N		74.8	N/A
pB27_A-97	3p	Mus musculus - Mast1	..2739	??	N		82.0	N/A
pB27_A-33	3p	Mus musculus - Mast1	..3395	??			98.3	A

Clone Name	Type Seq	Gene Name (Best Match)	Start..Stop (nt)	Frame	Sense	%Id 5p	%Id 3p	PBS
pB27_A-82	3p	Mus musculus - Mast1	..3619	??			96.9	A
pB27_A-93	5p/3p	Mus musculus - Mast1	2700..3405	IF		98.3	95.6	A
pB27_A-42	5p/3p	Mus musculus - Mast1	2700..3405	IF		98.3	84.9	A
pB27_A-12	5p/3p	Mus musculus - Mast1	2739..3395	IF		99.4	95.0	A
pB27_A-28	5p/3p	Mus musculus - Mast1	2739..3395	IF		99.5	96.3	A
pB27_A-36	5p/3p	Mus musculus - Mast1	2739..3409	IF		97.5	96.9	A
pB27_A-71	5p/3p	Mus musculus - Mast1	2739..3406	IF		99.3	95.5	A
pB27_A-125	5p/3p	Mus musculus - Mast1	2739..3416	IF		97.6	96.5	A
pB27_A-32	5p/3p	Mus musculus - Mast1	2745..3392	IF		92.2	97.8	A
pB27_A-157	5p/3p	Mus musculus - Mast1	2745..3392	IF		94.2	91.3	A
pB27_A-26	5p/3p	Mus musculus - Mast1	2757..3619	IF		96.2	86.8	A
pB27_A-15	5p/3p	Mus musculus - Mast1	2781..3339	IF		99.5	99.5	A
pB27_A-52	5p/3p	Mus musculus - Mast1	2781..3339	IF		99.6	99.3	A
pB27_A-64	5p/3p	Mus musculus - Mast1	2784..3495	IF		99.3	97.1	A
pB27_A-183	5p/3p	Mus musculus - Mast1	2784..3495	IF		97.5	95.0	A
pB27_A-24	5p/3p	Mus musculus - Mast1	2832..3382	IF		99.6	95.0	A
pB27_A-34	5p/3p	Mus musculus - Mast1	2832..3506	IF		96.6	87.9	A
pB27_A-40	5p	Mus musculus - Mast1	2832	IF		97.4		A
pB27_A-140	5p/3p	Mus musculus - Mast1	2832..3747	IF		92.7	91.9	A
pB27_A-182	5p/3p	Mus musculus - Mast1	2832..3747	IF		87.5	90.3	A
pB27_A-156	5p/3p	Mus musculus - Mast1	2832..3747	IF		93.6	95.5	A
pB27_A-51	5p/3p	Mus musculus - Mast1	2835..3397	IF		99.6	99.6	A
pB27_A-46	5p/3p	Mus musculus - Mast1	2835..3397	IF		99.6	97.1	A
pB27_A-57	5p/3p	Mus musculus - Mast1	2835..3392	IF		99.5	97.0	A
pB27_A-116	5p/3p	Mus musculus - Mast1	2835..3397	IF		99.5	99.3	A
pB27_A-127	5p/3p	Mus musculus - Mast1	2835..3397	IF		99.3	98.8	A
pB27_A-161	5p/3p	Mus musculus - Mast1	2883..2290	IF		88.9	60.3	A
pB27_A-101	5p/3p	Mus musculus - Mast2 var2	3249..4046	IF		95.8	95.4	B
pB27_A-108	5p/3p	Mus musculus - Mast2 var2	3249..4046	IF		94.3	91.9	B
pB27_A-114	5p/3p	Mus musculus - Mast2 var2	3306..3880	IF		85.8	76.9	B
pB27_A-136	3p	Mus musculus - Mpdz	..6018	??			92.3	B
pB27_A-163	5p/3p	Mus musculus - Mpdz	4812..5684	IF		89.1	89.8	B
pB27_A-175	5p/3p	Mus musculus - Mpdz	4812..5684	IF		95.2	95.3	B
pB27_A-60	5p/3p	Mus musculus - Mpdz	5043..6018	IF		96.4	91.7	B
pB27_A-5	5p/3p	Mus musculus - Mpdz	5313..6059	IF		96.8	95.7	B
pB27_A-18	3p	Mus musculus - Ptpn4	..2250	??			90.4	D
pB27_A-9	5p/3p	Mus musculus - Ptpn4	1482..2418	IF		89.4	95.1	D
pB27_A-111	5p/3p	Mus musculus - Shank1	1740..2626	IF		82.2	80.1	B
pB27_A-122	5p/3p	Mus musculus - Shank1	1749..2350	IF		88.4	83.6	B
pB27_A-109	5p/3p	Mus musculus - Shank1	1749..2350	IF		93.8	88.1	B
pB27_A-134	5p/3p	Mus musculus - Shank1	1803..2470	IF		94.5	96.4	B
pB27_A-77	5p/3p	Mus musculus - Shank1	1911..2468	IF		95.7	94.5	B
pB27_A-119	5p/3p	Mus musculus - Zfp521	234..1272	IF		92.9	92.8	E
pB27_A-69	5p/3p	Mus musculus - activin receptor-interacting protein2	-73..507	IF		99.1	88.5	C
pB27_A-6	5p/3p	Mus musculus - activin receptor-interacting protein2	-70..551	IF		99.5	97.0	C
pB27_A-41	3p	Mus musculus - GenMatch GI:23271896	..632	??			95.6	N/A
