# Supplementary material for: Csmd2 Is a Synaptic Transmembrane Protein that Interacts with PSD-95 and Is Required for Neuronal Maturation
Source: eNeuro. 2019 May 7;6(2):ENEURO.0434-18.2019. doi: 10.1523/ENEURO.0434-18.2019 (PMC6506821; doi:10.1523/ENEURO.0434-18.2019)

Figure 8-2. Yeast 2-hybrid Prey Fragment Analysis. Schematic representations of information on bait and prey structural, functional and interaction domains. Selected Interaction Domain (SID) is the amino acid sequence shared by all prey fragments matching the same reference protein. SIDs often correspond to an identified structural or functional domain.

HYBRiGENiCS SERVICES

DomSight: AMB_RP1_hgx3854v1 vs. Mouse Adult Brain_RP1 (24 Dec 2014)

(Bait plasmid(s): hgx3854v1_pB27)

Bait fragment
SID fragment
Pfam or SMART domain
Transmembrane domain (TMHMM)
Coiled-coil domain (Ncoils)
Signal peptide (SignalPHMM)

SID: Selected Interaction Domain
It is the amino acid sequence shared by all prey fragments matching the same reference protein. SIDs have been found in numerous cases to correspond to an identified structural or functional domain.

Legend

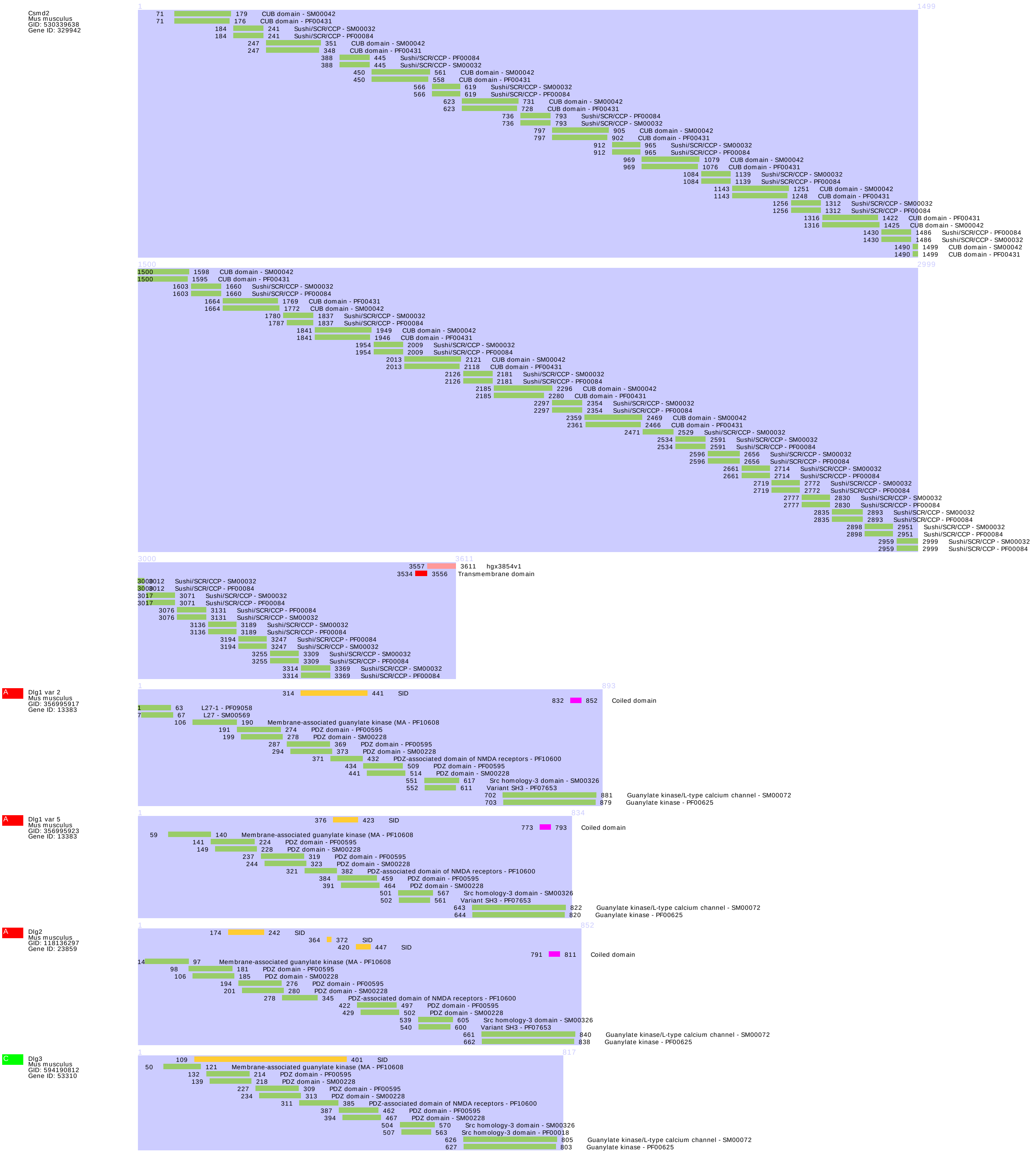

DomSight: AMB_RP1_hgx3854v1 vs. Mouse Adult Brain_RP1 (24 Dec 2014)
(Bait plasmid(s): hgx3854v1_pB27)

Bait fragment
SID fragment
Pfam or SMART domain
Transmembrane domain (TMHMM)
Coiled-coil domain (Ncoils)
Signal peptide (SignalPHMM)

SID: Selected Interaction Domain
It is the amino acid sequence shared by all prey fragments matching the same reference protein. SIDs have been found in numerous cases to correspond to an identified structural or functional domain.

Legend

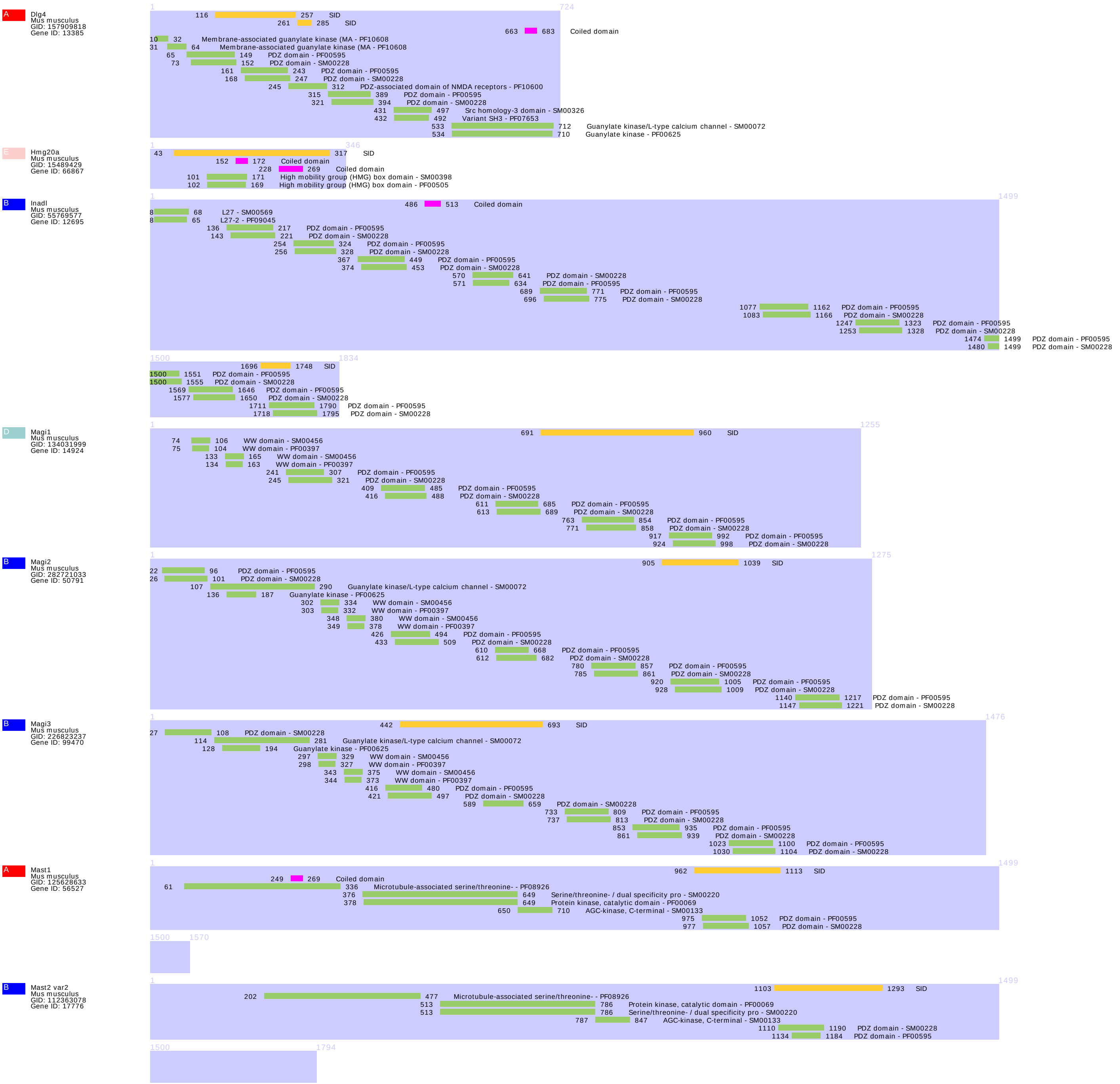

DomSight: AMB_RP1_hgx3854v1 vs. Mouse Adult Brain_RP1 (24 Dec 2014)

(Bait plasmid(s): hgx3854v1_pB27)

Bait fragment
SID fragment
Pfam or SMART domain
Transmembrane domain (TMHMM)
Coiled-coil domain (Ncoils)
Signal peptide (SignalPHMM)

SID: Selected Interaction Domain
It is the amino acid sequence shared by all prey fragments matching the same reference protein.
SIDs have been found in numerous cases to correspond to an identified structural or functional domain.

Legend

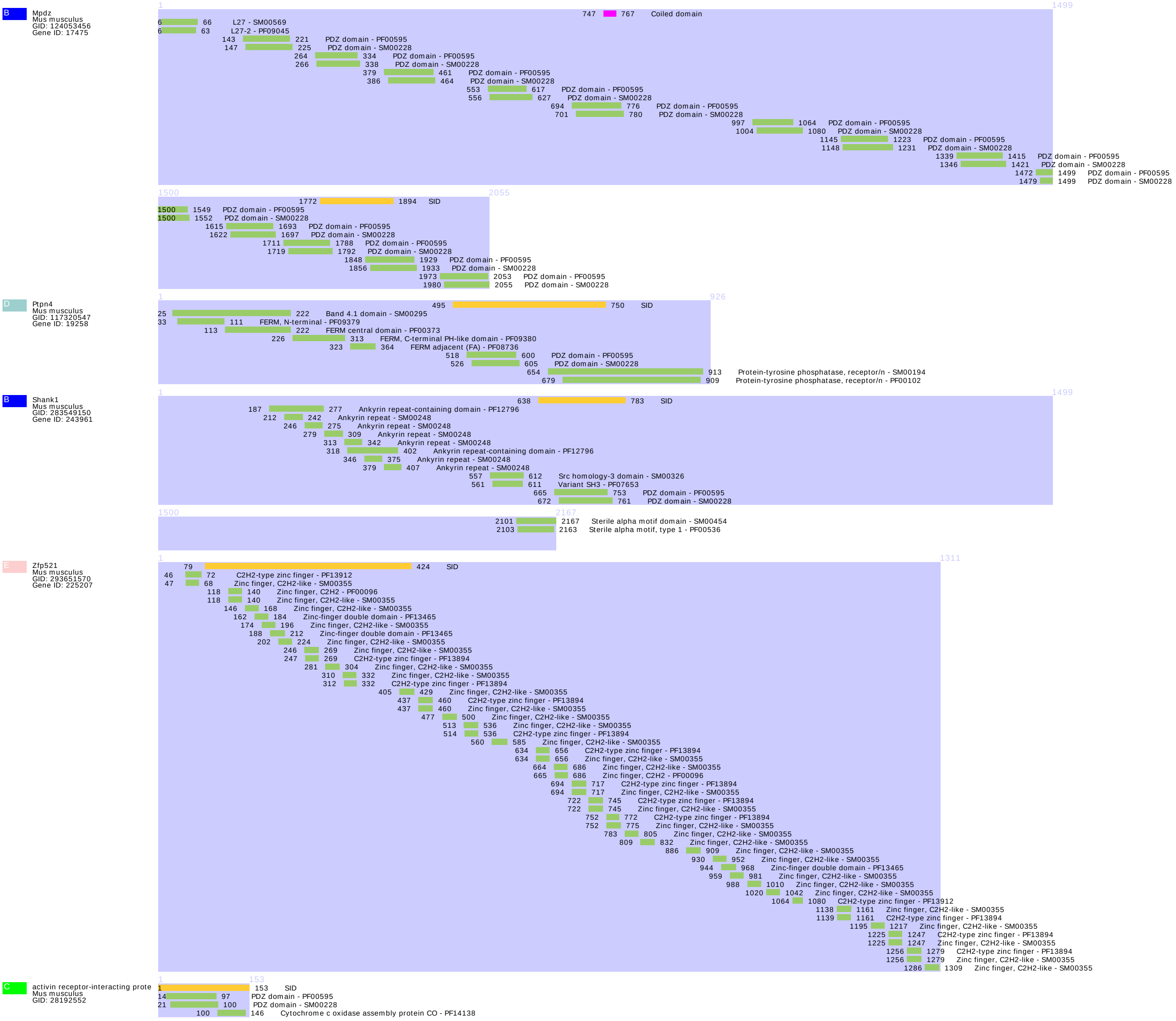

Supplement: Extended Data Figure 8-2 — Yeast 2-hybrid Prey Fragment Analysis. Schematic representations of information on bait and prey structural, functional and interaction domains. Selected Interaction Domain (SID) is the amino acid sequence shared by all prey fragments matching the same reference protein. SIDs often correspond to an identified structural or functional domain. Figure 8-2, PDF file. [file sup_enu-eN-NWR-0434-18-s02.pdf]
